# MacroH2A1.1 as a crossroad between epigenetics, inflammation and metabolism of mesenchymal stromal cells in myelodysplastic syndromes

**DOI:** 10.1038/s41419-023-06197-x

**Published:** 2023-10-18

**Authors:** C. Giallongo, I. Dulcamare, S. Giallongo, A. Duminuco, D. Pieragostino, M. C. Cufaro, A. M. Amorini, G. Lazzarino, A. Romano, N. Parrinello, M. Di Rosa, G. Broggi, R. Caltabiano, M. Caraglia, M. Scrima, L. S. Pasquale, M. S. Tathode, G. Li Volti, R. Motterlini, F. Di Raimondo, D. Tibullo, G. A. Palumbo

**Affiliations:** 1 Department of Medical, Surgical Sciences and Advanced Technologies “G.F. Ingrassia”, University of Catania, Catania, Italy; 2 Division of Hematology, AOU Policlinico, Catania, Italy; 3 Department of General Surgery and Medical-Surgical Specialties, University of Catania, Catania, Italy; 4 Department of Innovative Technologies and Medicine & Odontoiatry, University G. D’Annunzio, Chieti-Pescara, Italy; 5 Analytical Biochemistry and Proteomics Laboratory, Center for Advanced Studies and Technology (CAST), “G. d’Annunzio” University of Chieti-Pescara, Chieti, Italy; 6 Department of Biomedical and Biotechnological Sciences, University of Catania, Catania, Italy; 7 Departmental Faculty of Medicine and Surgery, UniCamillus-Saint Camillus International University of Health and Medical Sciences, Rome, Italy; 8 Department of Precision Medicine, University of Campania “Luigi Vanvitelli”, Naples, Italy; 9 Laboratory of Precision and Molecular Oncology, Biogem Scarl, Institute of Genetic Research, Ariano Irpino, Italy; 10 Faculty of Health, University Paris Est Créteil, INSERM, IMRB, Créteil, France

**Keywords:** Proteomics, Epigenetics

## Abstract

Ineffective hematopoiesis is a hallmark of myelodysplastic syndromes (MDS). Hematopoietic alterations in MDS patients strictly correlate with microenvironment dysfunctions, eventually affecting also the mesenchymal stromal cell (MSC) compartment. Stromal cells are indeed epigenetically reprogrammed to cooperate with leukemic cells and propagate the disease as “tumor unit”; therefore, changes in MSC epigenetic profile might contribute to the hematopoietic perturbations typical of MDS. Here, we unveil that the histone variant macroH2A1 (mH2A1) regulates the crosstalk between epigenetics and inflammation in MDS-MSCs, potentially affecting their hematopoietic support ability. We show that the mH2A1 splicing isoform mH2A1.1 accumulates in MDS-MSCs, correlating with the expression of the Toll-like receptor 4 (TLR4), an important pro-tumor activator of MSC phenotype associated to a pro-inflammatory behavior. MH2A1.1-TLR4 axis was further investigated in HS-5 stromal cells after ectopic mH2A1.1 overexpression (mH2A1.1-OE). Proteomic data confirmed the activation of a pro-inflammatory signature associated to TLR4 and nuclear factor kappa B (NFkB) activation. Moreover, mH2A1.1-OE proteomic profile identified several upregulated proteins associated to DNA and histones hypermethylation, including S-adenosylhomocysteine hydrolase, a strong inhibitor of DNA methyltransferase and of the methyl donor S-adenosyl-methionine (SAM). HPLC analysis confirmed higher SAM/SAH ratio along with a metabolic reprogramming. Interestingly, an increased LDHA nuclear localization was detected both in mH2A1.1-OE cells and MDS-MSCs, probably depending on MSC inflammatory phenotype. Finally, coculturing healthy mH2A1.1-OE MSCs with CD34^+^ cells, we found a significant reduction in the number of CD34^+^ cells, which was reflected in a decreased number of colony forming units (CFU-Cs). These results suggest a key role of mH2A1.1 in driving the crosstalk between epigenetic signaling, inflammation, and cell metabolism networks in MDS-MSCs.

## Introduction

Myelodysplastic syndromes (MDS) are a heterogeneous group of malignant disorders affecting the hematopoietic stem progenitor cell (HSPC) compartment [1]. Although recent advances in the field, existing treatments are generally not efficient [2]. The stromal cell compartment, where mesenchymal stromal cells (MSCs) are located, contribute to the typical MDS bone marrow (BM) microenvironment [3–5], as reported on different mice models [6–8]. MSC aberrations are driven by inflammatory triggers inducing BM failure and impairing hematopoietic stem cell (HSPC) functions, in a microenvironment already characterized by increased TGFβ [9, 10]. Furthermore, MDS-MSCs exhibit a premature replicative senescence contributing to the impairment of their stromal support [5, 11]. MSC dysfunctions are also depicted by epigenetic dysregulations [11, 12], as DNA methylation aberrations. Since MDS-MSCs display a hypermethylated DNA profile [13, 14], hypomethylating agents like azacytidine improve MSCs function [15]. Interestingly epigenetic marks can be amplified and transmitted to differentiated progeny; therefore, they are perpetuated as a crucial pathogenetic mechanism [16].

Accumulating evidence supports the contribution of histone variants in this context [17]. Here, we focus on the non-canonical histone H2A macroH2A1 (mH2A1), usually deleted in a subset of MDS patients [18]. Interestingly, mH2A1 correlates with the establishment of an heterochromatic state, as it also works in synergy with DNA methylation in repressing p16 [19]. The mRNA encoding for mH2A1 generates two splicing isoforms, mH2A1.1, harboring the peculiarity to bind ADP-ribose, and mH2A1.2 [18]. The two isoforms behave differently depending on the cellular context [20–24]. Interestingly, helicase U2AFY^S34F^ mutation in HSPCs leads to decreased mH2A1.1 levels, eventually impairing erythroid differentiation and granulomonocytic differentiation [25].

To date, the effects of mH2A1 on the inflammatory status of stromal cells has not been interrogated; therefore, we evaluated its role in patients’ MDS-MSCs. Furthermore, ectopic macroH2A1.1 overexpression in a healthy MSCs in vitro model reshapes their inflammatory and metabolic profiles, eventually resulting in an impairment of their hematopoietic support capacity. Overall, our findings identified the splicing isoform mH2A1.1 as an oncohistone orchestrating a pro-tumorigenic inflammatory status in MSCs associated to epigenetics and metabolic rewiring potentially contributing to ineffective hematopoiesis in MDS.

## Material and methods

### Patients and healthy donor bone marrow samples

After written informed consent (Azienda Ospedaliero-Universitaria Policlinico “G.Rodolico-San Marco”, n. 54/2022/PO), BM samples were collected from patients with diagnosis of MDS (*n* = 41) and age-matched healthy controls (HC; *n* = 17). Clinical data of patients included in this study are shown in Table 1. After MDS diagnosis, patients were stratified by evaluation of their hemoglobin and platelets value, absolute neutrophil count, rate of bone marrow blasts, and cytogenetics into five class-risk (very low, low, intermediate, high, and very high), based on the Revised International Prognostic Scoring System (IPSS-R) for Myelodysplastic Syndromes [26]. We further grouped patients into 2 different classes: lower-risk patients with IPSS-R ≤ 4.5 (very low, low, and intermediate) and higher-risk patients with IPSS-R > 4.5 (high, and very high).

**Table 1:** Clinical characteristics of patients included in the study.

	Low risk	High risk
	*N* = 26	*N* = 15
Age, years (IQR)	70 (63–79)	73 (70–77)
Gender		
Female, *n (%)*	11 (42)	7 (47)
Male*, n (%)*	15 (58)	8 (53)
Type of MDS		
MDS with single lineage dysplasia, *n (%)*	4 (15)	/
MDS with multilineage dysplasia, *n (%)*	21 (81)	4 (26)
MDS-EB-1, *n (%)*	1 (4)	7 (47)
MDS-EB-2, *n (%)*	/	4 (27)
Therapy-related, *n* (%)	1 (4)	1 (7)
Median blood count at diagnosis		
Hb, g/dL (IQR)	9.4 (8.3–10.9)	8.2 (7.6–8.6)
Platelets, 10^*3*^/mmc (IQR)	156 (98–279)	81 (23–255)
WBC, 10^*3*^/mmc (IQR)	3.5 (2.4–6.1)	3.5 (2.5–4.8)
Neutrophils, 10^*3*^/mmc (IQR)	2.8 (0.9–5.7)	1.3 (0.8–1.7)
Cytogenetics		
Normal karyotype, *n* (%)	20 (80)	10 (67)
Altered karyotype, *n* (%)	5 (20)	5 (33)
IPSS-R risk class		
Very low, *n* (%)	4 (16)	/
Low, *n* (%)	14 (56)	/
Intermediate, *n* (%)	7 (28)	7 (47)
High, *n* (%)	/	6 (40)
Very high, *n* (%)	/	2 (13)

*IQR* interquartile range, *MDS-EB* MDS with excess of blasts, *Hb* hemoglobin, *WBC* white blood cells count, *IPSS-R* Revised International Prognostic Scoring System.

### Immunohistochemistry

Immunohistochemical analysis was performed as already described [27]. Detailed method is available in the Supplementary material and methods.

### MSC collection, cell cultures, and treatments

MSCs from HC and MDS patients were obtained after density gradient centrifugation on Ficoll of BM samples [28]. Commercially available stromal cell line HS-5 was used. Details of cell cultures and transfection methods are described in Supplementary material and methods. 5-azacytidine was obtained from Sigma-Aldrich (St. Louis, MO, USA). Cell transfection was carried out using commercially available mH2A1.1- and mH2A1.2-overexpressing vectors as already described (Supplementary Fig. 1; Twist Bioscience, South San Francisco, CA, USA) [29].

### Immunofluorescence assay

Immunofluorescent analysis was performed as previously described [30]. Details are reported in the Supplementary material and methods.

### qPCR

Quantitative PCR was performed as previously described [27]. Primers used in this study and qPCR details are reported in the Supplementary material and methods.

### HPLC

Metabolic analysis was assessed after deproteinization of cell samples as previously described [31]. Detailed protocols are presented in the Supplementary material and methods.

### Western blot

Histone or nuclear proteins were isolated using respectively the Histone and nuclear Extraction Kit (ab113476 and ab113474, Abcam, Milan, Italy). Detailed methods are available in the Supplementary material and methods.

### RNA-seq

RNA-seq analysis was performed as previously described [32]. Details are described in the Supplementary material and methods.

### Proteomics

Proteomics was performed as previously described [33]. Detailed protocols are presented in the Supplementary material and methods.

### β-Galactosidase activity

Details are presented in the Supplementary material and methods.

### Flow cytometry

Details of Flow cytometry analysis were described in the Supplementary material and methods.

### Isolation of healthy BM CD34+ cells and Colony-forming unit cell (CFU-C) assay

Human BM CD34+ cells (obtained from healthy donors; *n* = 4) were selected using MiniMacs (Miltenyi Biotec, Bologna, Italy). Detailed information about co-culture experiments and CFU-C assay are reported in the Supplementary material and methods.

### In-cell Western assay

The In-cell Western assay was performed as previously reported [27]. Details are described in Supplementary material and methods.

### Statistical analysis

All statistics were performed as previously described using GraphPad Prism (version 5.00 for Mac, GraphPad Software, San Diego, CA, USA) [27]. Functional Enrichment Analysis (EA) was performed using the web utility, STRING (https://string-db.org/) [34]. The STRING was also used for building the weighted proteins networks commonly modulated, rendered by CorelDRAW2020 (Corel Corporation, Ottawa, Ontario, Canada). In order to perform t-Sne analysis, we used Morpheus, (https://software.broadinstitute.org/morpheus). The coordinates T1 and T2 obtained by Morpheus were used in GraphPad) in order to represent the distribution of significantly expressed proteins graphically. A p-value < 0.05 was considered to indicate a statistically significant difference between experimental and control groups.

## Results

### Expression of mH2A1 in tumor microenvironment of MDS patients

**Fig. 1:**
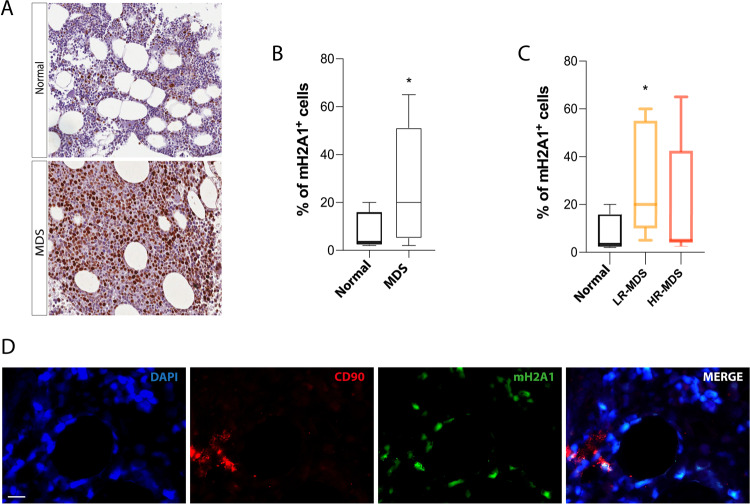
MH2A1 is overexpressed in MDS microenvironment. **A** Representative immune-histochemical image of mH2A1 in a control (top panel) and a MDS (bottom panel) BM slide. **B** The percentage of mH2A1^+^ cells was semi-quantitatively calculated comparing age-matched normal controls with MDS (**C**) or LR-MDS and HR-MDS patients. **D** Representative pictures of mH2A1 expression in MSCs, identified as CD90^+^ cells, in BM biopsies isolated from MDS patients. Scale bar: 10 µm. Bars indicate the standard error means. *p < 0.05.

To assess the levels of mH2A1 within the tumor microenvironment, we analyzed its expression in BM biopsies from healthy controls (*n* = 4) and MDS patients (22 of 41 enrolled in the study) by immunohistochemistry. A positive expression was observed in 19 of 22 cases (13.6%) whereas a negative expression was found in 3 of 22 cases (13.6%). Among the 3 negative biopsy specimens, one belonged to a patient with del(5q) MDS. MH2A1 staining was weak, moderate, and strong in 4 (18.1%), 7 (31.8%), and 8 (36.3%) cases, respectively (Supplementary Table 1). In healthy control specimens, the intensity of staining was moderate and strong in 1 and 3 of 4 cases, respectively. Evaluating the percentage of mH2A1 positive cells, we found a higher expression in MDS specimens compared to controls (25.65 ± 22.38% vs 7.25 ± 8.53% of controls, *p* < 0.05; Fig. 1A, B). Among the analyzed MDS specimens, 11 patients were classified as lower-risk (LR) MDS while 11 as higher-risk (HR) MDS. Compared to controls, only LR-MDS cases had a significant increased expression of mH2A1 (28.45 ± 21.52%, *p* < 0.05 compared to controls; Fig. 1C), while HR-MDS showed a wide range of protein expression, from low to high levels of positive cell percentages (with a mean value of 22.22 ± 24.22%). The Spearman correlation test depicted a linear correlation between the percentage of mH2A1 positive cells and the biopsy’s histological cellularity (*r* = 0.223, *p* < 0.05; Supplementary Table 2 and Supplementary Fig. 2). The highest incidence of cellularity was observed in the biopsy specimens of LR-MDS (45.4%) compared to HR-MDS (18.1%) (Supplementary Table 3), thus explaining the lack of significant difference in mH2A1 expression between HR-MDS and healthy control specimens. Immunofluorescence analysis revealed that MDS-MSCs were among the mH2A1 positive cells (Fig. 1D), prompting us to better investigate its potential role as a valid candidate characterizing the phenotype of these cells.

### mH2A1.1 expression in MDS-MSCs is associated to a pro-inflammatory TLR4-primed phenotype

**Fig. 2:**
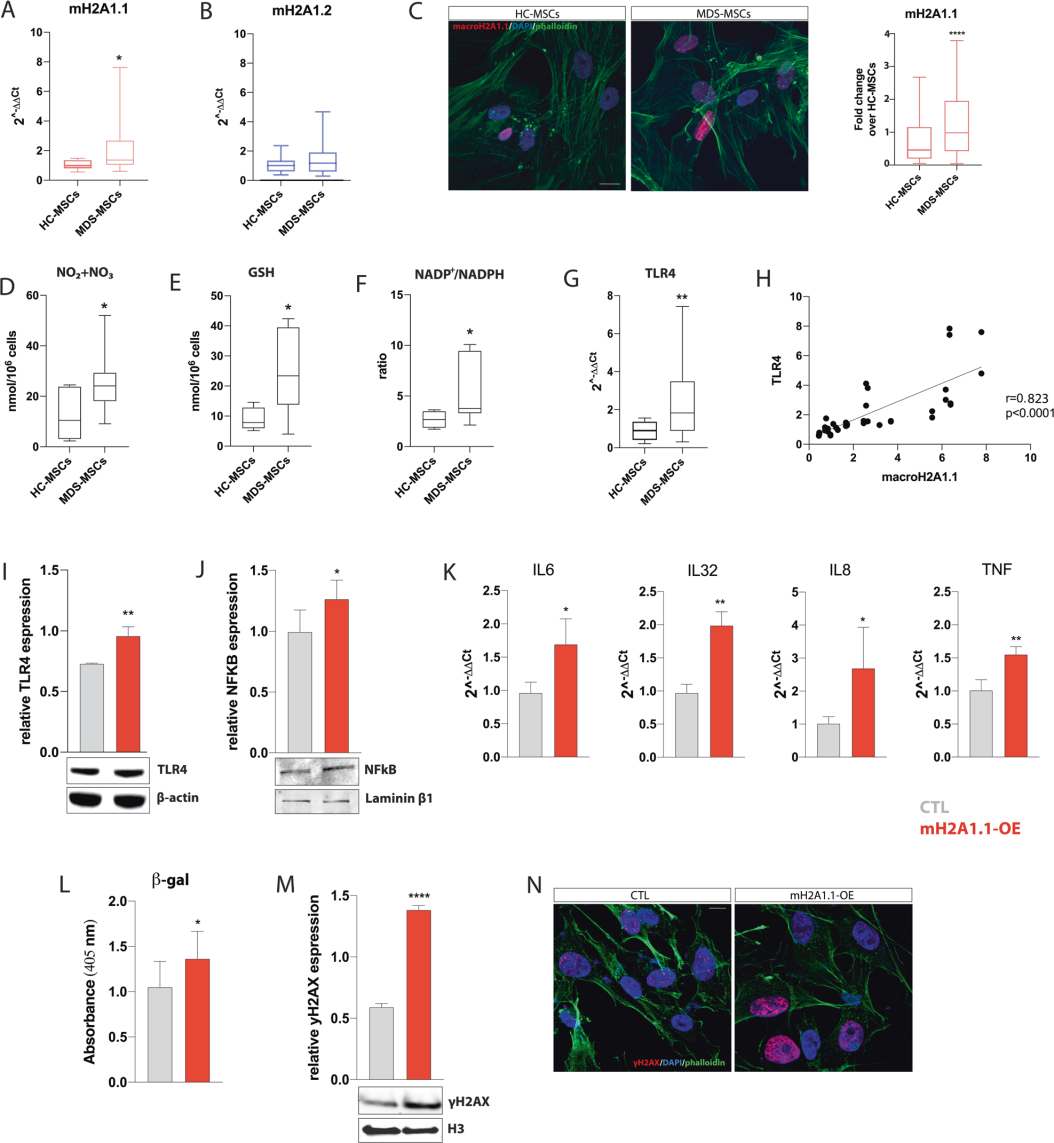
Upregulation of mH2A1.1 in MDS-MSCs directly correlates with a pro-inflammatory TLR4-primed phenotype. **A**, **B** Gene expression analysis of mH2A1 splicing isoforms on MSCs from MDS patients and age-matched healthy controls. B2M was used as housekeeping gene. **C** Representative pictures of MSCs from a healthy donor (left panel) and a MDS patient (right panel) stained for mH2A1.1. Scale bar: 20 µm. The protein MFI was analyzed on a total of 2 HC- and 4 MDS-MSCs; quantification was represented as fold change over HC-MSCs. **D** The sum of nitrites and nitrates, (**E**) GSH concentration and (**F**) NADP^+^/NADPH ratio were quantified by HPLC analysis in deproteinized HC- and MDS-MSCs. **G** TLR4 gene expression analysis in HC- and MDS-MSCs. B2M was used as housekeeping gene. **H** TLR4-mH2A1.1 gene expression correlation analysis in MDS-MSCs. **I**, **J** Western blot analysis of TLR4 and nuclear NFkB in HS-5 cells after 72 h from starting transfection. β-actin and laminin β1 proteins were used as total protein loading reference. The optical band density was measured using Scion Image software. **K** Expression of inflammatory genes by qPCR. B2M was used as housekeeping gene. Calculated value of 2^^−ΔΔCt^ in CTL was 1. **L** β-gal activity in CTL and mH2A1.1-OE. **M** Analysis of γH2AX expression by western blot. H3 was used as loading control. **N** Representative immunofluorescence images of CTL and mH2A1.1-OE for γH2AX. Scale bar: 10 µm. Data are presented as mean ± SD of three independent experiments. *p < 0.05; **p < 0.01; ****p < 0.0001.

To further assess mH2A1 accumulation within MSCs in MDS BM microenvironment, we analyzed the expression of the two splicing isoforms mH2A1.1 and mH2A1.2 in healthy and MDS-MSCs. Quantitative RT-PCR revealed a significant increased expression of mH2A1.1 in MDS-MSCs (*n* = 22; 20 LR- and 2 HR-MDS) compared to HC-MSCs (*n* = 12; *p* < 0.05; Fig. 2A). No significant difference was observed in mH2A1.2 expression (Fig. 2B). The higher expression of mH2A1.1 was confirmed by immunofluorescence in MSCs from 4 MDS patients (3 LR- and 1 HR-MDS; *p* < 0.0001 vs HC-MSCs (*n* = 2); Fig. 2C).

Furthermore, HLPC analysis on MDS-MSCs (*n* = 13; 9 LR- and 4 HR-MDS) showed an increase of the sum of nitrites and nitrates (NO_2_ + NO_3_), GSH level and NADP^+^/NADPH ratio compared to HC-MSCs (*n* = 5) (*p* < 0.05; Fig. 2D–F), suggesting that these cells are adapted to an inflammatory microenvironment. This is paralleled by the increased expression of Toll-like receptor 4 (TLR4) (*p* < 0.01; Fig. 2G), a key marker of pro-inflammatory MSCs [35]. Importantly, our analysis identified a positive correlation between mH2A1.1 and TLR4 expression (*r* = 0.823; *p* < 0.0001; Fig. 2H). Therefore, we aimed to address whether mH2A1.1 over-expression could affect TLR4 signaling. For this, we ectopically overexpressed mH2A1.1 in HS-5 (mH2A1.1-OE) cells using a specific vector (Supplementary Fig. 3A). We observed a significant increase in both TLR4 and nuclear NFkB expression in mH2A1.1-OE cells (*p* < 0.01; Fig. 2I, J) compared to control cells (CTL) transfected with the empty vector. The activation of a mH2A1.1-mediated pro-inflammatory response was also confirmed by the increase of pro-inflammatory genes upon transfection (Fig. 2K). However, the activation of TLR4 pathway was not exclusive for mH2A1.1; indeed, its activation was also observed after mH2A1.2 induction (Supplementary Fig. 3B–D). In agreement, a significant reduction of TLR4 levels was found after the whole mH2A1 silencing (Supplementary Fig. 3E, F), corroborating the existence of a mH2A1/TLR4 axis in stromal cells.

As mH2A1 is a critical component of the positive feedback loop regulating senescence-associated secretory phenotype (SASP) [36], we next investigated the potential role of mH2A1.1 in the activation of this phenotype in healthy MSCs. MH2A1.1-OE cells showed higher senescence-associated β-galactosidase (β-gal) activity (*p* < 0.05) and γH2AX expression (*p* < 0.0001) compared to CTL (Fig. 2L–N). These data suggest that upregulation of mH2A1.1 in MDS-MSCs contributes to the activation of a pro-inflammatory TLR4-primed phenotype alongside SASP activation.

### mH2A1.1 overexpression changes the proteomic and transcriptomic profile of MSCs

**Fig. 3:**
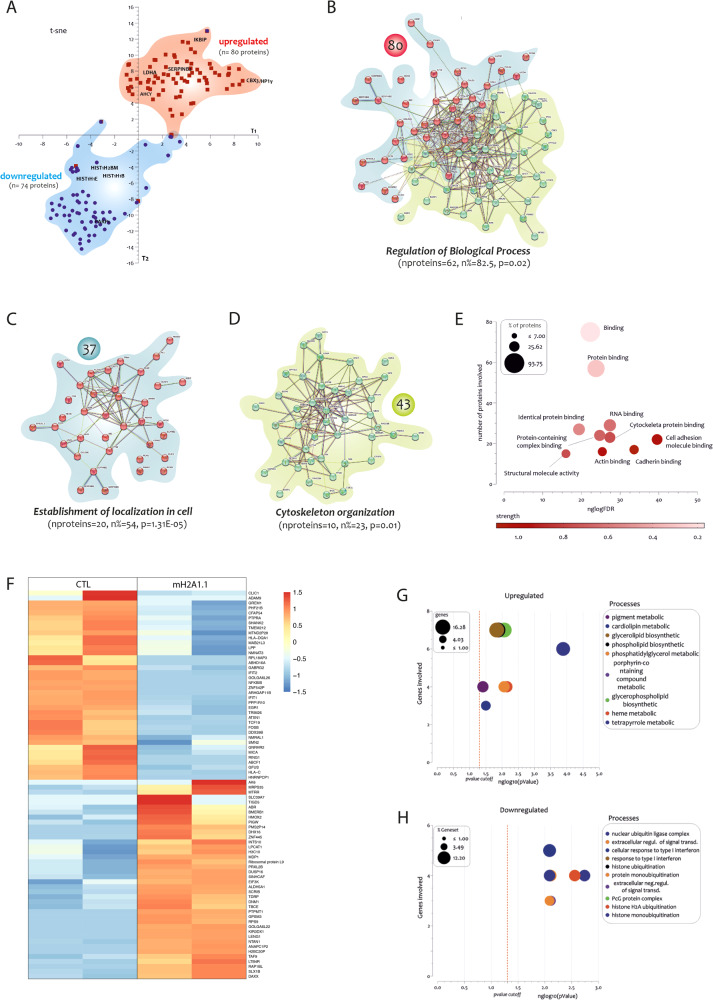
Proteomic and transcriptomic analysis of HS-5 cells overexpressing mH2A1.1. **A** Two-dimensional t-SNE map showed two distribution groups: in red the 80 upregulated proteins (in bold the IKBIP, CBX3, SERPINB2, LDHA, and AHCY belonging to the first group), and in blue the 74 downregulated proteins (in bold the HIST1H2BM, HIST1H1B, HIST1H1E, and PARP1 proteins belonging to group 2). **B** Clustering the upregulated 80 proteins is possible to highlight two main Clusters: (**C**) Cluster 1 consisting of 37 proteins and (**D**) Cluster 2 of 43 proteins. **E** The 80 proteins are involved in various biological processes such as cell adhesion molecule binding, cadherin binding, RNA binding, cytoskeletal protein binding, actin binding, protein containing complex binding, protein binding, binding, identical protein binding, and structural molecule activity. The *p*-value is intended as a False Discovery Rate (FDR). The FDR is the rate at which features called significantly are truly null. An FDR of 5% means that, among all features called significant, 5% of these are truly null. A *p*-value threshold (alpha) of 0.05 yields an FDR of 5% among all truly null features. **F** Heatmap of RNA-seq transcriptome analysis indicating differentially expressed genes between CTL and mH2A1.1-OE cells sorted by logFC. **G**, **H** GO analysis was performed on 40 upregulated and 38 downregulated genes in mH2A1.1-OE cells compared to CTL. The genes involved in the Biological Process are depicted as percentages on the y-axis and represented using a bubble chart, where the size of the bubbles corresponds to the number of genes involved. The FDR was calculated and presented as neglog10(pvalue) on the x-axis. *P*-values < 0.05 were considered statistically significant (neglog10(pvalue) > 1.30).

To further investigate the role of mH2A1.1 in the reprogramming of MSC phenotype, we performed proteomics analysis in mH2A1.1-OE and CTL cells finding 493 and 553 individual proteins that were identified and quantified in these two cell types, respectively. By carrying out a distribution analysis of the significantly modulated proteins, 80 were found to be upregulated and 74 down-regulated in mH2A1.1 (compared to CTL), as shown in the two-dimensional t-SNE map of Fig. 3A, thereby highlighting two distribution groups. As confirmed by western blot analysis (Supplementary Fig. 4A), poly (ADP-ribose) polymerase 1 (PARP1) was among the downregulated proteins; mH2A1.1 has the exclusive ability to interact with PARP1 and restrict its overactivation [37] and these results validate our experimental approach. Moreover, upregulation of mH2A1.1 in HS-5 cells caused a significant downregulation of the histone H2B type 1-M (HIST1H2BM) and of two non-canonical linker histone H1 isoforms, H1.4 (HIST1H1E) and H1.5 (HIST1H1B). The analysis of the upregulated proteins confirmed the occurrence of a pro-inflammatory phenotype in mH2A1.1-OE cells as showed by the significant increase of the level of the inhibitor of NFkB interacting protein (IKBIP), serpinB8, serpinB2, and serpinB6 compared to CTL. Among the upregulated proteins, we also identified two additional proteins that are involved in epigenetic remodeling: CBX3/HP1γ, a core component of heterochromatin protein 1, and adenosylhomocysteinase (AHCY), an enzyme catalyzing the reversible break of S-Adenosylhomocysteine (SAH) [38]. Additional experiments were performed to confirm their upregulation in mH2A1.1-OE cells (Supplementary Fig. 4B, C).

Significant upregulated proteins were uploaded into STRING database in order to evaluate Protein-Protein interactions (PPI). By showing a significant PPI enrichment *p*-value < 1.0 × 10^−16^, 62 out of the 80 proteins were involved in the “regulation of biological processes” (*n*% = 82.5, FDR = 0.02; Fig. 3B). Moreover, considering the knowledge on PPI, the 80 upregulated proteins were established in two main Clusters. The first cluster consisted of 37 proteins in red, 20 of which played a specific role in the “establishment of localization in cell” (*n*% = 54, FDR = 1.31^−05^; Fig. 3C). The second cluster consisted of 43 proteins of which 10 involved in “cytoskeleton organization” process (*n*% = 23, FDR = 0.01; Fig. 3D). In addition, functional enrichment analysis of the 80 upregulated proteins revealed how they were involved in various biological processes among which the first significant 10 were “cell adhesion molecule binding” (FDR = 1.27 × 10^−12^), “cadherin binding” FDR = 7.74E-11), “RNA binding” (FDR = 5.93E-09), “cytoskeletal protein binding” (FDR = 5.93 × 10^−09^), “actin binding” (FDR = 2.29 × 10^−08^), “protein containing complex binding” (FDR = 3.68 × 10^−08^), protein binding (FDR = 7.07 × 10^−08^), binding (FDR = 1.99 × 10^−07^), “identical protein binding” (FDR = 1.49 × 10^−06^), and, finally, “structural molecule activity” (FDR = 1.55 × 10^−^^05^), as shown in the bubble plot of Fig. 3E built according to the FDR value and the number of involved proteins.

The analysis of mH2A1.1-OE transcriptomic profile identified 40 up- and 38 down-regulated genes (Fig. 3F and Supplementary Tables 5 and 6). The GO analysis of the significantly upregulated genes in mH2A1.1-OE cells compared to CTL revealed that the most upregulated biological processes were related to hemoglobin metabolism. These processes included tetrapyrrole metabolic process (FRD = 0.00013, genes=6), heme metabolic process (FRD = 0.007, genes=4), and porphyrin-containing compound metabolic process (FRD = 0.0085, genes=4) (Fig. 3G). Conversely, the GO analysis of the significantly downregulated genes highlighted those associated with histone ubiquitination. The main ones among these were histone monoubiquitination (FRD = 0.0018, genes = 4), histone H2A ubiquitination (FRD = 0.0027, genes = 4), and histone ubiquitination (FRD = 0.0081, genes = 4) (Fig. 3H). Interestingly, upregulated genes included methionine synthase reductase (MTRR) (Fig. 3F and Supplementary Table 5), which encodes an enzyme responsible for methionine synthase activation in mammals [39], thus contributing to methionine synthesis and DNA methylation reactions.

Taken together, our data reveal a scenario in which mH2A1.1 ectopic expression reshapes MSCs proteomic profile, eventually affecting several factors that might be involved in rewiring MSCs epigenetic profile.

### mH2A1.1 increases the methylation index and rewires energetic metabolism of MSCs

**Fig. 4:**
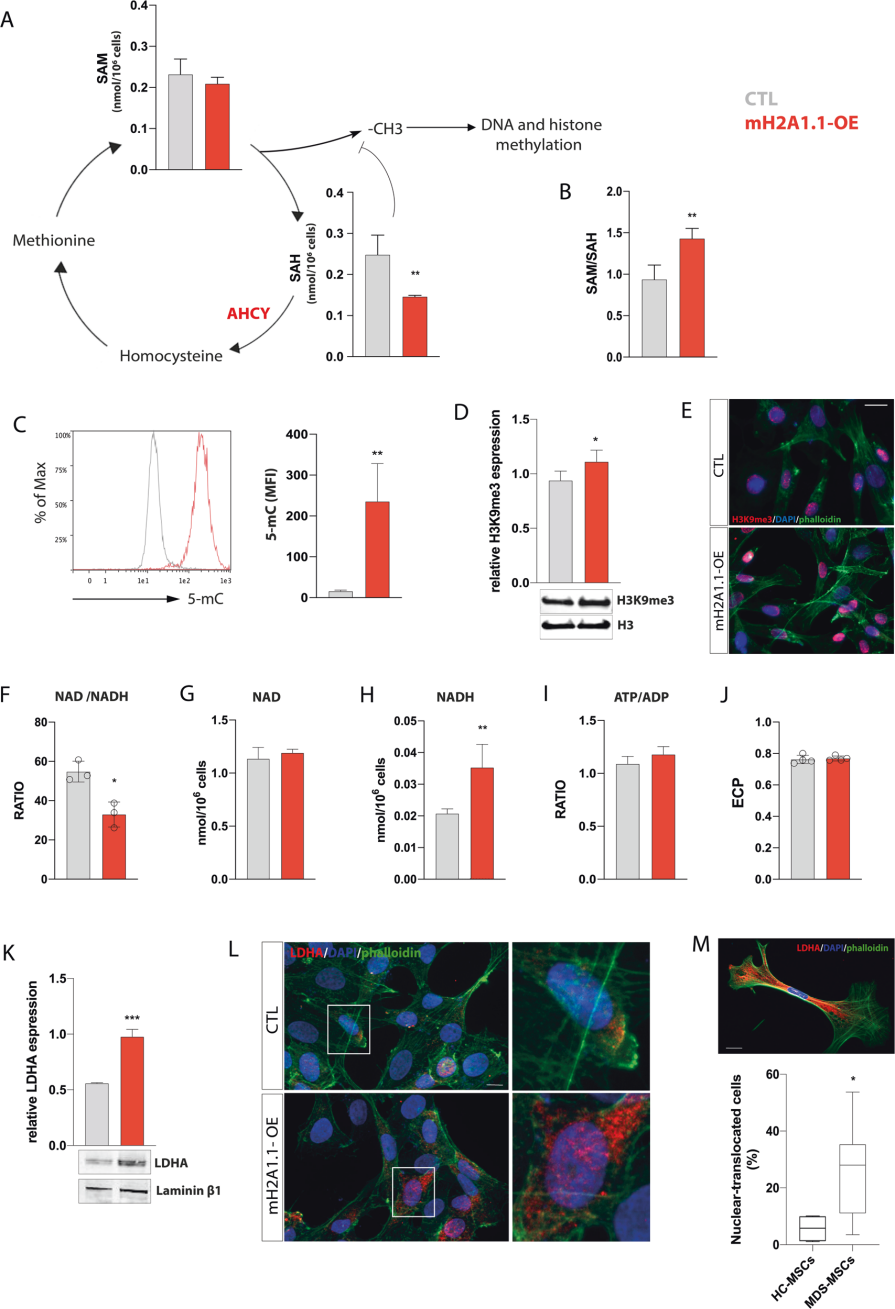
MH2A1.1 overexpression increases chromatin methylation index promoting a glycolytic profile. **A** SAM and SAH amounts were measured in mH2A1.1-OE cells and CTL 72 h upon transfection by using HPLC method. **B** SAM/SAH ratio quantification. **C** Flow cytometry analysis for 5-mC 72 h post transfection. A representative histogram showing the overlay of 5-mC-MFI for CTL and mH2a1.1-OE cells is shown. **D** Western blot analysis of H3K93me in HS-5 cells after 72 h from starting transfection. H3 protein was used as total protein loading reference. For analysis, the optical density of the bands was measured using Scion Image software. **E** Representative immunofluorescence images of CTL and mH2A1.1-OE for H3K9me3. Scale bar: 20 µm. **F**–**J** NAD^+^, NADH, ATP and ADP concentrations were calculated by HPLC analysis in CTL and mH2A1.1-OE cells. ECP was calculated as follows: ATP + 1/2 ADP/ATP + ADP + AMP. **K** Western blot analysis of nuclear LDHA. Laminin β1 protein was used as total protein loading reference. **L** Representative pictures of CTL and mH2A1.1-OE cells stained for LDHA. Scale bar: 20 µm. Quantification of the protein MFI in the nucleus was analyzed. **M** Analysis of the number of cells with nuclear LDHA in MDS-MSCs patients. A representative picture of a MDS stromal cell with nuclear LDHA is shown. Scale bar: 20 µm. All the data are presented as means ± SD of three independent experiments. *p < 0.05; **p < 0.01; ***p < 0.001; ****p < 0.0001.

Our proteomic analysis provides the first evidence on adenosylhomocysteinase (AHCY) overexpression in mH2A1.1-OE cells. This data prompted us to examine the levels of S-adenosyl-methionine (SAM) and SAH in these cells. While SAM levels were not affected, SAH resulted significantly downregulated in mH2A1.1-OE compared to CTL (*p* < 0.01; Fig. 4A), overall corroborating the above-described AHCY upregulation. As expected, the SAM/SAH ratio was thus higher in mH2A1.1-OE compared to CTL (*p* < 0.01; Fig. 4B), suggesting an increased in chromatin methylation in these cells. To confirm these data, we subsequently assessed the levels of 5-mC by flow cytometry. We detected an increase of 5-mC mean fluorescence intensity (MFI) in mH2A1.1-OE cells compared to CTL (23.547 ± 92.7 vs 15.8 ± 2.4; *p* < 0.01; Fig. 4C). Furthermore, western blot and immunofluorescence analyses also confirmed a higher amount of trimethylated histone H3 at lysine 9 (H3K9me3) (Fig. 4D, E), wherewith CBX3/HP1γ is recruited with high affinity.

Moreover, the proteomic approach revealed a marked increase in lactate dehydrogenase A (LDHA) (Fig. 3A), corroborated by the transcriptomic upregulation of Adenylate Kinase 6 (AK6) (Fig. 3F and Supplementary Table 5), encoding a key LDHA modulator, therefore promoting its activation [40]. For this reason, we sought to evaluate the metabolic profile of mH2A1.1-OE cells. MH2A1.1 overexpression resulted in a significant change of 3 metabolites: NADH, NADPH, and UDP-glucose (Supplementary Fig. 4D and Fig. 4H). The increased amount of NADH caused a decrease of NAD^+^/NADH ratio in mH2A1.1-OE cells compared to CTL (*p* < 0.05), while ATP/ADP ratio did not show any significant difference (Fig. 4F, G, I). These data suggest that a possible glycolytic shift in mH2A1.1-OE cells was not dependent on energy state alterations, as also supported by the lack of changes in energy charge potential (ECP) (Fig. 4J). It has recently been reported, on the other hand, that LDHA might localize into the nucleus sensing intracellular reactive oxygen species (ROS) accumulation [41]. Compared to control, mH2A1.1-OE cells showed increased nuclear LDHA (Fig. 4K, L) associated to higher levels of ROS (*p* < 0.01; Supplementary Fig. 4E). This observation biased us to investigate LDHA localization also in primary MDS-MSCs. Our results showed an increased percentage of nuclear LDHA in MDS-MSCs (*n* = 7; 5 LR- and 2 HR-MDS) compared to HC-MSCs (*n* = 4) (*p* < 0.05; Fig. 4M). These data provide increasing evidence on the outstanding role played by mH2A1.1 in rewiring MSCs methylome profile, eventually also affecting their metabolic landscape.

### Overexpression of mH2A1.1 in MSCs attenuates CD34^+^ cell number and progenitor function

**Fig. 5:**
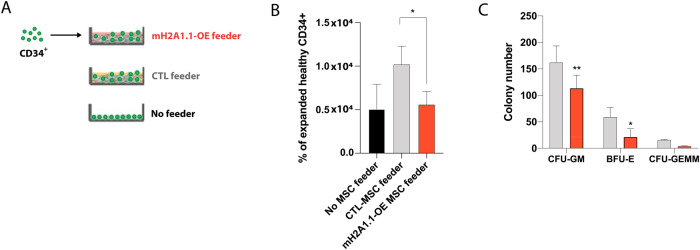
Overexpression of mH2A1.1 in healthy MSCs impairs their hematopoietic support capacity. **A** Schematic representation of the experimental design. Healthy CD34^+^ cells were expanded in co-culture for 5 days with HC-MSCs overexpressing or not mH2A1.1. **B** Flow cytometric analysis showing the significant reduction in the number of CD34^+^ cells from cocultures with mH2A1.1-OE MSCs compared to control MSCs. **C** Hematopoietic CFC potential of healthy CD34^+^ cells derived from cocultures with mH2A1.1-OE or control MSCs. Frequencies for CFU-BFU, CFU-GM, and CFU-GEMM were calculated after 14 days after co-cultures were stopped. Data represent mean ± SD of two independent experiments performed in triplicate. *p < 0.05; **p < 0.01.

In MDS patients, the majority of CD34^+^ HSPCs are in direct contact with MSCs [42]. Therefore, we next performed co-culture experiments with healthy MSCs transfected to overexpress mH2A1.1 and healthy CD34^+^ cells (Fig. 5A) aiming to address whether the upregulation of this histone variant in stromal cells might affect HSPCs. The co-culture on mH2A1.1-OE MSCs resulted in significant reduced number of CD34^+^ cells (*p* < 0.05 vs CTL feeder; Fig. 5B), reflected in a lower number of CFU-GM and BFU-E (*p* < 0.01 and *p* < 0.05, respectively, compared to CTL feeder; Fig. 5C). Interestingly, the co-culture of CD34^+^ cells with mH2A1.2-OE MSCs did not change neither their proliferative capacity nor their progenitor function (Supplementary Fig. 5A, B), confirming mH2A1.1 exclusive involvement in MSC hematopoietic support. Together, these data display the essential role played by mH2A1.1 in stromal cells, where it seems to affect functions of CD34^+^ cells.

### Azacytidine treatment negatively regulates mH2A1.1/TLR4 axis

**Fig. 6:**
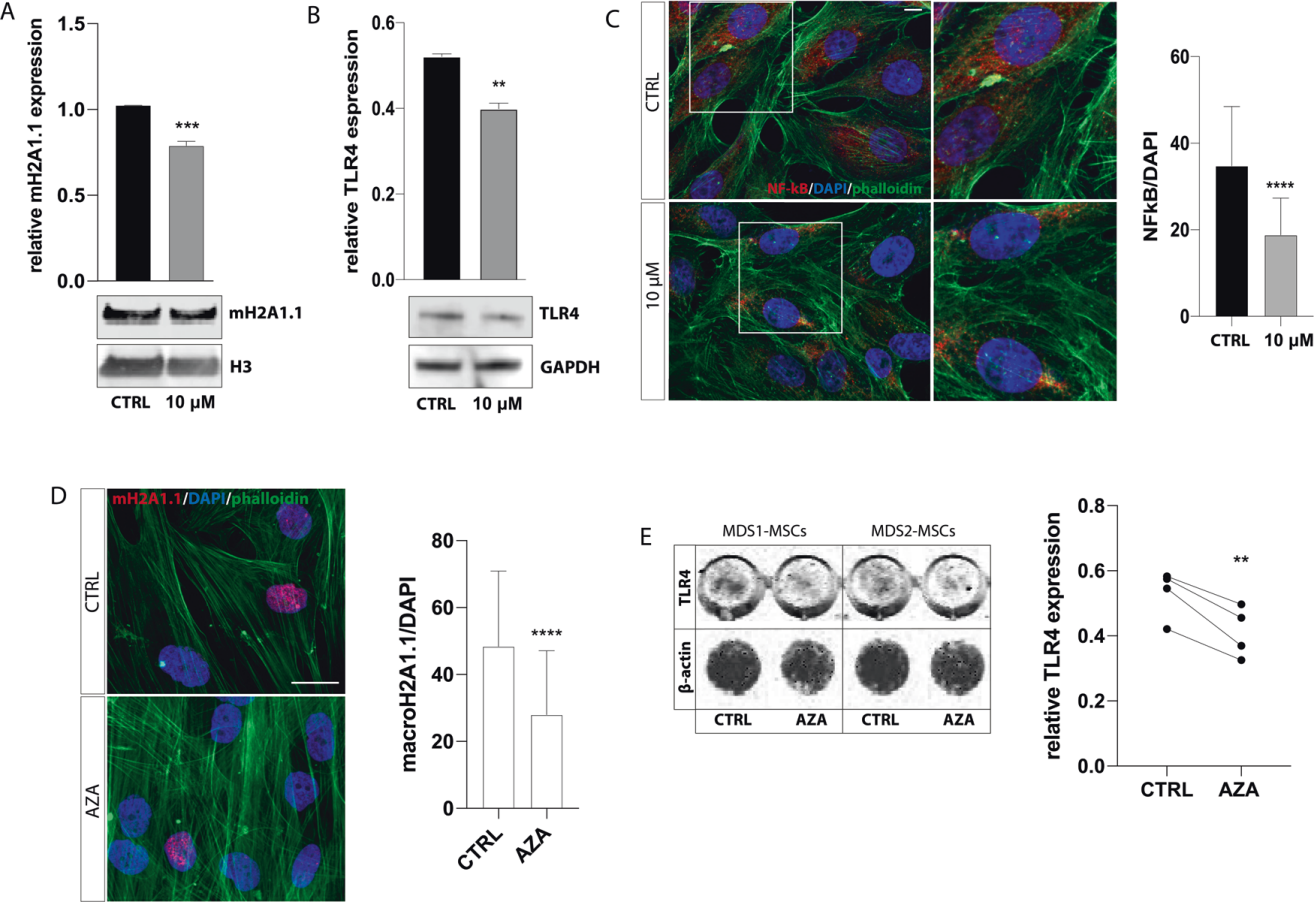
MH2A1.1/TLR4 axis is downregulated by azacytidine. **A**, **B** Western blot analysis of mH2A1.1 and TLR4 in HS-5 cells 48- and 72 h after AZA treatment, respectively. As total protein loading reference, H3 and GAPDH protein were used. The optical density of the bands was measured using Scion Image software. **C** Representative pictures of NFkB staining in HS-5 control (upper panel) or treated with AZA 10 μM (bottom panel) for 72 h. Scale bar: 10 µm. Quantification of the protein MFI in the nucleus (right histogram). **D** Representative pictures of mH2A1.1 staining in MDS-MSCs treated ex vivo with AZA 10 μM. Scale bar: 20 µm. Quantification of the protein MFI in the nucleus for 4 patients was calculated and graphed. **E** Representative image of In-cell Western assay for MSCs isolated from 2 MDS patients after exposure to AZA for 72 h and quantitative analysis of TLR4 expression after AZA treatment for 4 MDS patients (right histogram). Quantification of the protein was performed using β-actin protein as total protein loading reference and Scion Image software. All the data are presented as means ± SD of three independent experiments. *p < 0.05; **p < 0.01; ***p < 0.001; ****p < 0.0001.

As it has been reported that MSCs of myeloid neoplasms can be therapeutically targeted using epigenetic drugs such as azacytidine (AZA) [43], we finally treated HS-5 stromal cells for 72 h with AZA to evaluate its effect onto mH2A1.1/TLR4 axis. After 48 h, treated cells exhibited a significant reduction of mH2A1.1 levels (*p* < 0.001 compared to untreated cells; Fig. 6A), followed by a decreased expression of both TLR4 and nuclear NFkB after 72 h (respectively *p* < 0.01 and *p* < 0.0001; Fig. 6B, C). Corroborating this evidence, ex vivo treatment with AZA was able to reduce both mH2A1.1 and TLR4 protein levels also in MSCs from MDS patients (*n* = 4) (*p* < 0.0001 and *p* < 0.01 compared to control cells; Fig. 6D, E). These data suggest that the direct effect of AZA on HSPC-supporting capacity of MDS-MSCs [43, 44] may be in part dependent on its ability to regulate mH2A1.1 expression.

## Discussion

The histone variant mH2A1 is involved in repressive-chromatin establishment [45], associating with the Polycomb repressive complexes and their mark H3K27me3 [46]. While normal cells typically express both mH2A1 variants, mH2A1.1 expression is lost in many cancers, eventually unveiling its contribution as a tumor suppressor [21, 23, 47]. In MDS, downregulation of mH2A1.1 has been linked to mutations affecting U2AF1 [25, 48]. Furthermore, several MDS patients lack chromosome 5q, where H2AFY is located [49]. Here, we show that mH2A1 expression is increased in biopsy specimens from MDS patients, especially in LR-MDS. Among the BM microenvironment components, MSCs contribute to the accumulation of mH2A1 in MDS milieu, in particular of mH2A1.1 isoform. Moreover, our data demonstrate that these cells are characterized by an increased sum of nitrites and nitrates, GSH, and NADP^+^/NADPH ratio, suggesting their adaptation to an inflammatory status, confirmed also by TLR4 overexpression. The interplay between the inflammatory BM *milieu* and the acquisition of driver mutations in HSPCs is crucial for MDS pathogenesis [50]. TLR4 and other TLRs have been reported to be overexpressed in MDS-HSPCs [51, 52] and TLR4 expression was shown to correlate with CD34^+^ apoptosis [7]. Concerning MSCs, TLR4-primed stromal cells mostly elaborate pro-inflammatory mediators [35, 53]. In accordance with TLR4 upregulation in MDS-MSCs, a transcriptional landscape characterized by cellular stress and upregulated inflammatory molecules with a hematopoietic inhibitory effect in LR-MDS has been reported [54]. Interestingly, in MDS-MSCs we found a positive correlation between TLR4 and mH2A1.1, suggesting a link between epigenetic changes and establishment of MDS inflammatory phenotype. Therefore, we next confirmed mH2A1.1-mediated activation of TLR4 pathway using a transfected model. Interestingly, also the isoform mH2A1.2 is able to activate TLR4 indicating that its activation is not exclusive for mH2A1.1. MDS-MSCs have a senescent profile which promotes inflammation and reduces stem cell support capacity [55]. Consistent with this, mH2A1.1 upregulation causes SASP activation as demonstrated by the increase of β-gal activity and γH2AX expression.

The activation of the pro-inflammatory cascade observed in mH2A1.1-OE cells was further supported by proteomic analysis showing overexpression of serpinB8, serpinB2, serpinB6, and IKBIP. SerpinB2 orchestrates TLR4-activated macrophages cascade [56, 57], thus contributing to mH2A1.1-mediated TLR4 activation. On the other hand, IKBIP is a negative regulator of NFkB profoundly associated with extracellular matrix organization and cell-substrate adhesion [58]. Consistent with IKBIP overexpression, proteomic analysis revealed upregulation of factors involved in cytoskeleton organization and cell adhesion structures (vinculin, lamin-B1, Filamin A, αActinin-4 and 1, tubulin α, spectrin α chain, filamin C and cortactin) (dataset identifier PXD039866). We also reported downregulation of the histone H1.4, associated to a strong interferon response when depleted, thus corroborating mH2A1.1-induced inflammatory phenotype [59]. Moreover, mH2A-mediated heterochromatin establishment is orchestrated in synergy with lamin B [46, 47], as supported by our data reporting lamin-B1 as one of the prominent upregulated proteins in mH2A1.1-OE. Besides, we unveil the upregulation of epigenetic remodelers as AHCY and CBX3/HP1γ. AHCY is part of DNA methyltransferase 1 (DNMT1) interactome, responsible for the ex novo DNA methylation [60], a process generating SAH [61]. AHCY is the only mammalian enzyme hydrolyzing SAH, once recruited by DNMT1; therefore, its overexpression leads to the hypermethylation of the genome [62]. Accordingly, mH2A1.1-OE cells enhanced MTRR gene expression and exhibited an increased SAM/SAH ratio, overall indicating an augmented chromatin methylation index, also confirmed by enhanced 5mC levels. DNA methylation is related to histone modifications, as the lack of H3K4me3 and the presence of H3K9me3 [63, 64]. In mH2A1.1-OE, we found an accumulation of Cbx3/HP1γ, a member of the histone readers heterochromatin 1 family, in turn interacting with H3K9 methyl groups [65]. Accordingly, western blot analysis showed an increased level of transcriptional repression marker H3K9me3 in MSCs with mH2A1.1 upregulation.

Among the proteins upregulated in our model we found LDHA. This metabolism-epigenome crosstalk assists cells in the adaptation to their environment. Accordingly, the inflammatory MSC-licensing causes a metabolic shift enhancing glycolysis [66]. The elevated oxidative stress induces LDHA nuclear translocation, where it gains a noncanonical enzyme activity producing α-hydroxybutyrate (α-HB) and activating an antioxidant response by epigenetic modifications [41, 67]. Corroborating this, mH2A1.1-OE exhibited higher nuclear LDHA and increased ROS. Since it has been showed that nuclear LDHA binds DOT1L methyltransferase increasing histone methylation levels [41], this protein might contribute to the metabolic-epigenetic crosstalk. Notably, MDS-MSCs increase the number of LDHA-translocated cells, in agreement with their aberrant methylation [68, 69].

Given mH2A1.1-driven changes, we unveil that its overexpression impairs MSCs hematopoietic support capacity, which was not affected by the upregulation of mH2A1.2. These data agree with mH2A1.1-induced inflammatory profile, in turn responsible for the activation of pathways harboring an hematopoietic inhibitory effect [70, 71].

Recently, the effect of AZA on the hematopoietic niche has been shown [43]. Its efficiency is also biased by the stromal epigenetic landscape [43]. The aberrant MDS-MSCs methylation is indeed reverted in AZA-patients [68]. Our data on AZA-induced inhibition of mH2A1.1/TLR4 axis corroborate the notion that stromal gene networks influenced by AZA have a direct effect on the HSPC-supporting BM niche providing evidence on therapeutic potential of epigenetic treatment of MDS-MSCs.

Finally, our findings establish that mH2A1.1 is a key driver for the inflammatory, epigenetic, and metabolic regulation of MSC activity in MDS. The next challenge in the field is to understand how epigenetic changes, inflammatory signals, and metabolic signature co-ordinate to form a particular epigenotype leading to MDS-MSC dysfunctional phenotype.

### Supplementary information

Supplemental material

Original Data File

Original Data File
