# Supplementary material for: MacroH2A1.1 as a crossroad between epigenetics, inflammation and metabolism of mesenchymal stromal cells in myelodysplastic syndromes
Source: Cell Death Dis. 2023 Oct 18;14(10):686. doi: 10.1038/s41419-023-06197-x (PMC10584900; doi:10.1038/s41419-023-06197-x)
Supplement: Supplementary file 1 — Supplemental material [file 41419_2023_6197_MOESM1_ESM.pdf]

SUPPLEMENTARY MATERIAL AND METHODS

Immunohistochemistry

Deparaffinized slides underwent pretreatment with PBS followed by 0.2% biotin-PBS, each for 15 min at room temperature, and then rinsed for 20 min in order to obtain the reduction of the non-specific staining caused by endogenous biotin. The antigenic unmasking was obtained by microwave pretreatment. Subsequently the slides were incubated overnight at 4°C with rabbit polyclonal anti-macroH2A1 antibody (ab232602; Abcam, Milan, Italy) diluted 1:200 in PBS (Sigma, Milan, Italy). Sections were counterstained with hematoxylin, dehydrated, mounted (Zymed Laboratories, San Francisco, CA, USA), and observed with a light microscope (Carl Zeiss, Oberkochen, Germany). MacroH2A1 was considered as positive if brown chromogen was observed in the cellular nucleus. A semiquantitative method based on both the intensity of staining (IS) and the percentage of immunoreactive cells (Extent Score- ES) was applied to estimate the macroH2A1 immunoreactivity, as previously described [1]. In more detail, a 0-3 score was assigned to IS (0 = absent staining, 1 = weak staining, 2 = moderate staining, 3 = strong staining), while a 0–4 score was used for ES (<5%; 5–30%; 31–50%; 51–75%; >75%). A routinary visual estimation of BM cellularity was performed by assessing the marrow specimen at 40x, 100x and 400x and estimating the percentage cellularity as the total area of the specimen in which hematopoietic cells were visible, excluding bone spicules and blood vessels. Three classes of cellularity were identified, accordingly: low, moderate and high.

MSC collection and culturing conditions

MSCs and HS-5 cell line were cultured in Dulbecco's modified Eagle's medium (DMEM, Life Technologies, Milan, Italy) supplemented with 10% heat-inactivated FBS, 100 U/ml penicillin, 100 mg/ml streptomycin and 1% l-glutamine. Cultures were maintained at 37 °C and 5% CO2 and MSCs were processed within the third passage. Cultured BM-MSCs population was identified as CD45-/CD34-/CD73+/CD105+ using flow cytometry.

Cell transfections

Cells were seeded the day prior to transfection onto 6-well plates at the density of $4x10^5$ cells/well. After 24 h, cells were transfected with 0.8 µg scramble vector, mH2A1.1-overexpressing or mH2A1.2-overexpressing plasmid using TurboFectin Transfection Reagent (TF81001 Origene, Herford, Germany) according to the manufacturer protocol. After 48h cells were assayed for mH2A1.1 or mH2A1.2 overexpression. For siRNA experiments (sc-91790, Santa Cruz Biotechnology, Heidelberg, Germany), cells were transfected according to the manufacturer's instructions.

Immunofluorescence assay

Paraffin sections from biopsy specimens from MDS patients (n=4) were permeabilized using 0.3% Triton X and blocked to prevent nonspecific antibody binding using a 0,3% Triton X-10% NGS solution. The slides were then incubated overnight at 4 °C with the primary antibodies rabbit anti-macroH2A1 (ab232602, Abcam) and mouse anti-CD90 (ab181469; Abcam) in 0,3% Triton-X. Subsequently, cells were washed three times in PBS and then incubated for 1 h at room temperature with the appropriate combination of fluorescence conjugated secondary antibodies donkey polyclonal anti-rabbit Alexa Fluor 647 (A32849, Thermo Fisher Scientific) and subsequently with goat polyclonal anti-mouse Alexa Fluor 488 (A21247, Thermo Fisher Scientific).

For immunofluorescence analysis in HS-5 cells or primary MSCs, cells were fixed using 4% paraformaldehyde (PFA) prior to permeabilization. Primary antibodies against human macroH2A1.1 (mAb#12455), NFk-B (mAb#3033S) from Cell Signaling (Danvers, MA, USA), LDHA (ab52488), H3K9me3 (ab8898) from Abcam, yH2AX (sc-517348; Santa Cruz Biotechnology) and CBX3 (11650-2-AP, Proteintech, Manchester, UK) were used. After incubation with Alexa Fluor 647, phalloidin staining was performed using Phalloidin-iFluor 488 (ab176753, Abcam) at dilution 1:500 and incubated for 1 h at room temperature.

After washing, nuclei were counterstained with DAPI for 5 min, at room temperature. Slices were mounted with fluorescent mounting medium Permafluor (Thermo Fisher Scientific) and digital

images were acquired using a Zeiss Axio Imager Z1 Microscope with Apotome 2 system (Zeiss, Milan, Italy).

qPCR

The relative transcription of human genes mH2A1.1 (Fw: TTCACCCGACAAAACACTGAC; Rw: GAGTTCCAGGACAGCTTCCAC), mH2A1.2 (Fw: TCCTTGGCCAGAAGCTGAAC; Rw: GAGTTCCAGGACAGCTTCCAC), TLR4 (Fw: AAGCCGAAAGGTGATTGTTG; Rw: CTGAGCAGGGTCTTCTCCAC), IL6 (Fw: GAAAGCAGCAAAGAGGCACT; Rw: TTTCACCAGGCAAGTCTCCT), IL32 (Fw: TCTCGGCTGAGTATTTGTGCCA; Rw: AAGGAGCCAAGGCCTGAGATG), IL8 (Fw: ATGACTTCCAAGCTGGCCGTG; Rw: TGTGTTGGCGCAGTGTGGTC); TNF (Fw: GCCCATGTTGTAGCAAACCC; Rw: GGAGGTTGACCTTGGTCTGG) were analyzed using Brilliant III Ultra-Fast SYBR Green QPCR Master Mix (Agilent Technologies, Milan, Italy) and 7900HT Fast Real-Time PCR System (Thermo Fisher Scientific). For each sample, the relative expression level of the mRNA of interest was determined by comparison with the control housekeeping gene B2M (Beta-2-Microglobulin; Fw: AGCAGCATCATGGAGGTTTG; Rw: AGCCCTCCTAGAGCTACCTG) using the $2^{\wedge-\Delta\Delta Ct}$ method. To evaluate TLR4 and mH2A1.1 correlation, their $2^{\wedge-\Delta\Delta Ct}$ values were used.

HPLC

Metabolic analysis was assessed after deproteinization of cell samples as previously described [2]**.** After centrifugation, the organic solvent was removed from the deproteinizing mixture using two washings with chloroform. The upper aqueous phase obtained by centrifugation at the same conditions was then used for the HPLC analysis of low molecular weight metabolites. The simultaneous separation of 50 low molecular weight metabolites related to energy metabolism, oxidative/nitrosative stress and antioxidants and including high energy phosphates (ATP, ADP, GTP, GDP, UTP, UDP, CTP, and IMP), oxidized and reduced nicotinic coenzymes (NAD^+, NADH, $NADP^+$ and NADPH), glycosylated UDP-derivatives (UDP-galactose, UDP-glucose, UDP-N-acetyl-glucosamine and UDP-N-acetyl-galactosamine), S-adenosil metionina (SAM), reduced glutathione (GSH), nitrite and nitrate

were carried following slight modifications of previously established ion pairing HPLC methods [3,4]. The intracellular concentrations of S-adenosylhomocysteine (SAH) were analyzed as ortophtalaldehyde (OPA) derivatives using a different method with pre-column derivatization [5]. For all the aforementioned analyses, the HPLC apparatus consisted of a Surveyor System that was connected to a highly sensitive PDA diode-array detector (Thermo Fisher Scientific Italia, Rodano, Milan, Italy), equipped with a 5-cm light-path flow cell and set up to acquire signals between 200 and 400 nm wavelengths. The separation of the various compounds was carried out using a Hypersil C-18, 250 × 4.6 mm, 5 µm particle size column, provided with its own guard column (Thermo Fisher Scientific, Rodano, Milan, Italy). Data were acquired and analyzed by ChromQuest® software package provided by the HPLC manufacturer. Assignments and calculations of the aforementioned compounds in cell extracts were performed by comparing the retention times, absorption spectra, and areas of the peaks (calculated at the 260 nm wavelength for all compounds but GSH, nitrite and nitrate, which were calculated at the 206 nm wavelength and SAH calculated at 338 nm wavelength) of the chromatographic runs of mixtures containing known concentrations of ultrapure standards. All values were normalized to the number of cells and expressed as nmol/10^6 cells.

Western blot

Extracted proteins were loaded onto a 12% polyacrylamide gel Mini- PROTEAN TGXTM (BIO-RAD, Milan, Italy) followed by electrotransfer to nitrocellulose membrane Trans-Blot TurboTM (BIO-RAD) using Trans-Blot SE Semi-Dry Transfer Cell (BIO-RAD). Subsequently, membrane was blocked in chemiluminescent blocker (Millipore, Darmstadt, Germany) or in Odyssey Blocking Buffer (Licor, Milan, Italy) for 1 h at room temperature. After three washes, membrane were incubated with primary antibodies against human macroH2A1.1 (mAb#12455), NFk-B (mAb#3033S), mH2A1.2 (mAb#4827) from Cell Signaling, TLR4 (ab13867), H3K9me3 (ab8898), mH2A1 (ab232602), LDHA (ab52488), H3 (ab1791), GAPDH (ab181602), β-actin (ab8226), laminin β1 (ab109293), PARP1 (sc-8007) from Abcam, AHCY (sc-271389) and yH2AX (sc-517348) from Santa Cruz Biotechnology. Next day, after three washes, the membranes were incubated with

antimouse and anti-rabbit HRP-conjugated (Jackson, WestGrove, PA, USA) or Infrared antimouse IRDye800CW and antirabbit IRDye700CW secondary antibodies (Licor) for 1h at RT. Proteins bands were visualized with premixed ready-to-use chemiluminescent HRP detection reagent (Millipore) and the C-DiGit Blot Scanner (LI-COR Biosciences, Nebraska USA) or using Odyssey Infrared Imaging Scanner (Licor). The density of each band was quantified using ImageJ analysis software and normalized to protein levels of H3, β-actin, laminin β1 or GAPDH.

RNAseq

RNA of three independent experiments were run in triplicates for CTL and mH2A1.1-OE cells. Library preparation was performed using Illumina Stranded Total RNA Preparation protocol. Whole transcriptome paired-end sequencing was performed using Illumina NextSeq 550 sequencer.

Raw sequencing reads were assessed for quality check using FastQC v0.11.9. High quality reads were filtered having PHRED quality score cut off 30. Detailed sample wise read statistics is given in Supplementary Table 4. Also, filtered reads were screened for contamination associated with other sequences including vectors, adapters, and cross-species contamination if it exists, high quality reads were mapped against hg38 reference transcriptome to get transcript-level quantification for each sample using Salmon v1.8.0 in quant-based mode. Reference transcriptome index for Salmon was generated using cDNA sequences [GRCh38.p13 Ensembl release v109]. Principle Component Analysis (PCA) plot was derived using normalized log2 transformed counts for each sample to understand the clustering pattern within control and treated samples. Transcript level abundance is converted to gene-level abundance data using tximport R package v1.24.0. Gene level abundance is used to identify differentially expressed genes in H2A1.1 treated samples in comparison with control samples. Differential gene expression analysis was performed using DESeq2 v4.2 R package. From the total set of expressed RNAs, list of differentially expressed protein-coding genes were filtered using cut-off criteria's including FDR adjusted p-value <= 0.05 & log2Fold Change = |0.5|. Gene ontology (GO) analysis was conducted using the web tool GeneMANIA (http://genemania.org/) [6].

Proteomics

CTL and mH2A1.1-OE cells were lysed in buffer (UREA 6 M, Tris Base 100 mM, CHAPS 2%, Triton X 1%, dithiothreitol (DTT) 50 mM) and then the protein concentration was evaluated through Bradford assay (Bio-Rad, Hercules, CA, USA) in order to digest 30 µg of proteins. Specifically, proteins were separated by 10% SDS-PAGE to concentrate each cell line in a single band. Gel bands were digested according to in-gel digestion protocol by reducing the disulfide bonds with 10 mM DTT at 56°C for 1h, subsequently alkylated with 55 mM iodoacetamide at RT in the dark for 30 minutes, and then digested with trypsin overnight (Sigma-Aldrich, St. Louis, MO, USA). Peptides were eluted from gel pieces using acetonitrile from 50% to 100% in 0.1% of formic acid. Then, for protein label-free quantification, tryptic peptides from each sample were analyzed in triplicate with LC-MS/MS using the UltiMate™ 3000 UPLC (Thermo Fisher Scientific, Waltham, MA, USA) chromatographic system coupled to the Orbitrap Fusion™ Tribrid™ (Thermo Fisher Scientific) mass spectrometer, following the parameters described in Potenza et al. [7].

MaxQuant version 1.6.10.50 (Max-Planck Institute for Biochemistry, Martinsried, Germany) was used to process proteomics MS/MS raw data against the UniProt database (released 2020_06, taxonomy Homo Sapiens, 20,588 entries) in Andromeda peptide search engine. LFQ Intensities were used to quantify protein abundance in each sample in order to perform bioinformatics analysis with Perseus version 1.6.10.50 (Max-Planck Institute for Biochemistry, Martinsried, Germany). Processing parameters were set as already reported in our previous works [4,8,9]. STRING v. 11.5 was used for functional analysis to evaluate protein-protein interactions (PPI). The mass spectrometry proteomics data have been deposited to the ProteomeXchange Consortium via the PRIDE [10] partner repository with the dataset identifier PXD039866.

β-Galactosidase activity

β-Gal activity was assayed using beta-Galactosidase Assay Reagent, according to manufacturer instruction (#75705, Thermofisher Scientific). 72 h upon transfection, the media was discarded and cells were lysed using 500mL of M-PER mammalian protein extraction reagent (#78501,

Thermofisher Scientific) per well. Cells were incubated for 5 minutes at room temperature with a gentle shaking. In a microplate, 50mL of cell lysate was added to 50mL of beta-Galactosidase Assay Reagent. The plate was incubated for 30 minutes at 37oC. β-Gal activity was finally measured by absorbance at 405nm.

Flow cytometry

To evaluate the levels of 5-methylcytosine (5-mC), cells were washed twice with ice-cold PBS, resuspended with BD cytofix/cytoperm solution and incubated at 4°C for 20 min. Then, HCl (2M) was added for 5 min. After washing, cells were incubated with rabbit monoclonal anti-5mC antibody (ab214727, Abcam) at room temperature for 30 minutes. After washing, a donkey anti-rabbit IgG antibody (FITC) (GTX26798, CA, USA) was added to the samples for 20 min. The fluorescence was analyzed by flow cytometer MACSQuant Analyzer 10 (Miltenyi Biotec, Bologna, Italy). Samples incubated only with the anti-rabbit IgG antibody were used as negative controls.

The levels of reactive oxygen species (ROS) were detected using the 2′,7′-Dichlorodihydrofluorescein acetate (Sigma-Aldrich) and fluorescence intensity was measured according to the fluorescence detection conditions of FITC.

Culture of healthy BM CD34+ cells and Colony-forming unit cell (CFU-C) assay

For the co-culture setting, healthy MSCs (n=6) were transfected for 48h by empty vector, mH2A1.1 or mH2A1.2 plasmids before the experiment. The next day, MSCs were trypsinized and plated with healthy CD34+ cells at ratio 10:1 using Stem Cell Growth medium. Cultures were kept at 37°C in a 5% CO_2 incubator for 5 days until further analysis.

Colony-forming unit cell (CFU-C) assay

After co-culture, CD34+ cells were collected, counted by flow cytometry using flow count fluorophores (7547053, Beckman Coulter Life Sciences, Mylan, Italy). Then, 3000 cells were added to MethoCult^{TM} GF H4434 (StemCell Technologies, Cologne, Germany), which allows the growth of colonies from all three lineages. For every condition duplicate dishes were plated and kept at 37°C in a 5% CO_2 incubator for 2 weeks until colony counting.

Statistical analysis

All statistics were performed as previously described using GraphPad Prism (version 5.00 for Mac, GraphPad Software, San Diego, CA, USA) [11]. Functional Enrichment Analysis (EA) was performed using the web utility, STRING (https://string-db.org/) [12]. The STRING was also used for building the weighted proteins networks commonly modulated, rendered by CorelDRAW2020 (Corel Corporation, Ottawa, Ontario, Canada). In order to perform t-Sne analysis, we used Morpheus, (https://software.broadinstitute.org/morpheus). The coordinates T1 and T2 obtained by Morpheus were used in GraphPad) in order to represent the distribution of significantly expressed proteins graphically. A p-value < 0.05 was considered to indicate a statistically significant difference between experimental and control groups.

SUPPLEMENTAL REFERENCES

1. Broggi G, Lo Giudice A, Di Mauro M, et al. SRSF-1 and microvessel density immunohistochemical analysis by semi-automated tissue microarray in prostate cancer patients with diabetes (DIAMOND study). *Prostate.* 2021;81(12):882-892.
2. Giallongo C, Dulcamare I, Tibullo D, et al. CXCL12/CXCR4 axis supports mitochondrial trafficking in tumor myeloma microenvironment. *Oncogenesis.* 2022;11(1):6.
3. Tibullo D, Giallongo C, Romano A, et al. Mitochondrial Functions, Energy Metabolism and Protein Glycosylation are Interconnected Processes Mediating Resistance to Bortezomib in Multiple Myeloma Cells. *Biomolecules.* 2020;10(5).
4. Amorini AM, Giorlandino C, Longo S, et al. Metabolic profile of amniotic fluid as a biochemical tool to screen for inborn errors of metabolism and fetal anomalies. *Mol Cell Biochem.* 2012;359(1-2):205-216.
5. Amorini AM, Lazzarino G, Di Pietro V, et al. Severity of experimental traumatic brain injury modulates changes in concentrations of cerebral free amino acids. *J Cell Mol Med.* 2017;21(3):530-542.
6. Zuberi K, Franz M, Rodriguez H, et al. GeneMANIA prediction server 2013 update. *Nucleic Acids Res.* 2013;41(Web Server issue):W115-122.
7. Potenza F, Cufaro MC, Di Biase L, et al. Proteomic Analysis of Marinesco-Sjogren Syndrome Fibroblasts Indicates Pro-Survival Metabolic Adaptation to SIL1 Loss. *Int J Mol Sci.* 2021;22(22).
8. Falasca K, Lanuti P, Ucciferri C, et al. Circulating extracellular vesicles as new inflammation marker in HIV infection. *AIDS.* 2021;35(4):595-604.
9. Madonna R, Pieragostino D, Cufaro MC, et al. Ponatinib Induces Vascular Toxicity through the Notch-1 Signaling Pathway. *J Clin Med.* 2020;9(3).

10. Perez-Riverol Y, Bai J, Bandla C, et al. The PRIDE database resources in 2022: a hub for mass spectrometry-based proteomics evidences. *Nucleic Acids Res.* 2022;50(D1):D543-D552.
11. Barbato A, Giallongo C, Giallongo S, et al. Lactate trafficking inhibition restores sensitivity to proteasome inhibitors and orchestrates immuno-microenvironment in multiple myeloma. *Cell Prolif.* 2023:e13388.
12. Szklarczyk D, Gable AL, Lyon D, et al. STRING v11: protein-protein association networks with increased coverage, supporting functional discovery in genome-wide experimental datasets. *Nucleic Acids Res.* 2019;47(D1):D607-D613.

Supplementary Table 1. MH2A1 expression in biopsy specimens. Evaluation of the different staining of mH2A1 was semi quantitatively performed. Intensity of staining (IS) was graded on a 0–3 scale (0 = absent staining, 1 = weak staining, 2 = moderate staining, 3 = strong staining).

IS	HEALTHY	LR-MDS	HR-MDS	TOT
ABSENT	0	1	2	3
WEAK	0	2	2	4
MODERATE	1	3	4	7
STRONG	3	5	3	8

Supplementary Table 2. Relationship between the percentage of mH2A1 positive cells and cellularity in biopsy specimens of MDS patients. Five categories of percentage of mH2A positive cells (Extent Score, ES) were identified: <5%; 5–30%; 31–50%; 51–75%; >75%.

ES	LOW	MODERATE	HIGH
<5%	3	0	0
5-30%	5	2	0
31-50%	2	2	4
51-75%	1	0	4
>75%	0	0	0

$p<0.05$

Supplementary Table 3. Cellularity observed in LR-MDS and HR-MDS biopsy specimens.

CELLULARITY	LR-MDS	HR-MDS
LOW	5	6
MODERATE	1	3
HIGH	5	2

Supplementary Table 4. Sample wise read statistics of CTL and mH2A1.1-OE cells.

Sample Name	Sample type	Count of reads [R1]	Count of reads [R2]	Total no. of HQ reads [R1+R2]
CTL1_S1	Control Rep1	78,196,298	78,196,298	**156,392,596**
CTL2_S2	Control Rep2	59,687,644	59,687,644	**119,375,288**
CTL3_S3	Control Rep3	57,979,365	57,979,365	**115,958,730**
T1_S4	Treatment Rep1	66,022,621	66,022,621	**132,045,242**
T2_S5	Treatment Rep2	58,254,657	58,254,657	**116,509,314**
T3_S6	Treatment Rep3	57,812,063	57,812,063	**115,624,126**

Supplementary Table 5. All significantly upregulated genes in RNAseq experiment depicted in the heat map shown in Fig.3F are listed. Log2 fold changes, p-values and adjusted p-values are reported for each gene.

Genes Upregulated in mH2A1.1-OE vs CTL

ENSEMBL Gene ID	Gene Symbol	log2 Fold Change	p Value	Adjusted p Value
ENSG00000224399	SLC39A7	24.05329348	4.99E-07	1,90E-04
ENSG00000278016	TIGD5	22.81530081	1.86E-06	6,38E-04
ENSG00000085231	AK6	22.60009814	2.32E-06	7,75E-04
ENSG00000110536	PTPMT1	10.74468794	3.17E-13	4.75E-10
ENSG00000277808	LENG1	10.30708485	3.32E-12	2.58E-09
ENSG00000275323	RPS9	9.345568304	1.18E-09	7.08E-07
ENSG00000231259	ANAPC1P2	9.00345131	5.67E-09	3.24E-06
ENSG00000276033	KIR3DX1	8.732144326	1.71E-08	8.76E-06
ENSG00000213654	GPSM3	8.399793103	1.65E-07	7.35E-05
ENSG00000206486	DHX16	8.267184269	1.05E-06	3,88E-04
ENSG00000274404	GOLGA6L22	7.971723343	8.95E-07	3,35E-04
ENSG00000254893	RAP1BL	7.164623444	6.08E-05	1,29E-02
ENSG00000261716	H2BC20P	6.773855304	2,29E-04	3,70E-02
ENSG00000277125	PMS2P14	6.736161103	3,42E-04	4,98E-02
ENSG00000213920	MDP1	5.708823057	7.04E-09	3.84E-06
ENSG00000285053	TBCE	3.64149784	8.63E-12	6.28E-09
ENSG00000181625	SLX1B	3.565967164	6.35E-25	2.18E-21
ENSG00000157045	NTAN1	2.803902865	6.11E-36	4.89E-32
ENSG00000159842	ABR	1.897998444	1,54E-04	2,60E-02
ENSG00000277161	PIGW	1.545641374	1.02E-08	5.33E-06
ENSG00000206279	DAXX	1.415067614	1.56E-11	1.10E-08
ENSG00000112294	ALDH5A1	1.13504926	5.54E-05	1,18E-02
ENSG00000111266	DUSP16	1.118735639	6.89E-09	3.84E-06
ENSG00000106976	DNM1	1.108760562	5.11E-06	1,59E-03
ENSG00000180190	TDRP	1.021317852	2,74E-04	4,19E-02
ENSG00000285456	LTB4R	0.953131543	7.30E-05	1,51E-02
ENSG00000103415	HMOX2	0.857419796	2.86E-05	6,93E-03
ENSG00000153395	LPCAT1	0.773018583	2.36E-05	5,91E-03
ENSG00000166780	BMERB1	0.691735429	2,77E-04	4,21E-02
ENSG00000157870	PRXL2B	0.680552937	4.55E-05	1,03E-02
ENSG00000237550	Ribosomal protein L9	0.660720013	8.60E-11	5.74E-08
ENSG00000278828	H3C10	0.65814656	2,31E-04	3,70E-02
ENSG00000274287	SCRIB	0.634331986	5.84E-06	1,77E-03
ENSG00000061794	MRPS35	0.631624089	8.84E-05	1,76E-02
ENSG00000178982	EIF3K	0.617597084	2.06E-05	5,46E-03
ENSG00000283034	ZNF445	0.6011156	3.69E-05	8,68E-03
ENSG00000276463	TAF9	0.560209098	2.23E-05	5,69E-03
ENSG00000139146	SINHCAF	0.53108393	2.31E-05	5,85E-03
ENSG00000124275	MTRR	0.517364729	2,10E-04	3,40E-02
ENSG00000104613	INTS10	0.507832554	1,20E-04	2,24E-02

Supplementary Table 6. All significantly downregulated genes in RNAseq experiment depicted in the heat map shown in Fig.3F are listed. Log2 fold changes, p-values and adjusted p-values are reported for each gene.

Genes downregulated in mH2A1.1-OE vs CTL

ENSEMBL Gene ID	Gene Symbol	Log2 Fold Change	p Value	Adjusted p Value
ENSG00000282230	ADAM9	-24.30128267	3.80E-07	1,54E-04
ENSG00000233890	TCF19	-11.33126809	2.89E-06	9,51E-04
ENSG00000104825	NFKBIB	-11.04911623	5.45E-14	9.35E-11
ENSG00000231129	ABCF1	-9.889326804	1,45E-04	2,53E-02
ENSG00000286139	ARHGAP11B	-9.45545706	5.56E-10	3.43E-07
ENSG00000273756	GOLGA6L26	-8.239208869	3.04E-07	1.30E-04
ENSG00000240225	ZNF542P	-7.933724617	1.36E-06	4,89E-04
ENSG00000213442	RPL18AP3	-7.932302064	1.80E-05	5,07E-03
ENSG00000258900	HNRNPCP1	-7.824584981	4.53E-06	1,43E-03
ENSG00000224552	ABHD16A	-7.269720483	1,45E-04	2,53E-02
ENSG00000204227	RING1	-7.073344733	3,15E-04	4,72E-02
ENSG00000231225	MICA	-3.246332603	4.17E-05	9,53E-03
ENSG00000205571	SMN2	-3.031635494	7.58E-05	1,52E-02
ENSG00000230685	CLIC1	-2.877046663	2,41E-04	3,82E-02
ENSG00000204525	HLA-C	-2.30313405	3.29E-08	1.65E-05
ENSG00000235291	PPP1R10	-2.039392844	6.63E-49	7.96E-45
ENSG00000211451	GNRHR2	-1.827362457	1.71E-06	6,04E-04
ENSG00000278243	GFUS	-1.759110445	8.66E-13	1.15E-09
ENSG00000153406	NMRAL1	-1.604067698	7.25E-14	1.16E-10
ENSG00000166923	GREM1	-1.183245244	5.07E-13	7.16E-10
ENSG00000056487	PHF21B	-1.156661197	7.80E-06	2,31E-03
ENSG00000162105	SHANK2	-1.08751214	5.29E-05	1,14E-02
ENSG00000234127	TRIM26	-1.066463283	1.99E-10	1.29E-07
ENSG00000186329	TMEM212	-0.903948125	6.39E-05	1,33E-02
ENSG00000173212	MAB21L3	-0.857864266	3.88E-05	9,05E-03
ENSG00000188596	CFAP54	-0.836988456	2.70E-05	6,61E-03
ENSG00000230624	DDX39B	-0.821068358	2,56E-04	4,01E-02
ENSG00000113327	GABRG2	-0.78631642	1.21E-07	5.48E-05
ENSG00000163864	NMNAT3	-0.686284177	1,13E-04	2,16E-02
ENSG00000120738	EGR1	-0.647068209	2.77E-11	1.90E-08
ENSG00000124788	ATXN1	-0.633346378	2.70E-09	1.58E-06
ENSG00000228284	HLA-DQA1	-0.58840192	1,94E-04	3,21E-02
ENSG00000185745	IFIT1	-0.573852859	1.80E-07	7.85E-05
ENSG00000119922	IFIT2	-0.561608456	4.24E-12	3.18E-09
ENSG00000125740	FOSB	-0.554826962	4.87E-08	2.39E-05
ENSG00000145012	LPP	-0.533883229	1,93E-04	3,21E-02
ENSG00000225630	MTND2P28	-0.52073112	1,33E-04	2,35E-02
ENSG00000132670	PTPRA	-0.518143306	4.00E-05	9,24E-03

Supplementary Figure 1. Schematic representation of mH2A1.1 (**A**) and mH2A1.2 (**B**) overexpressing vector.

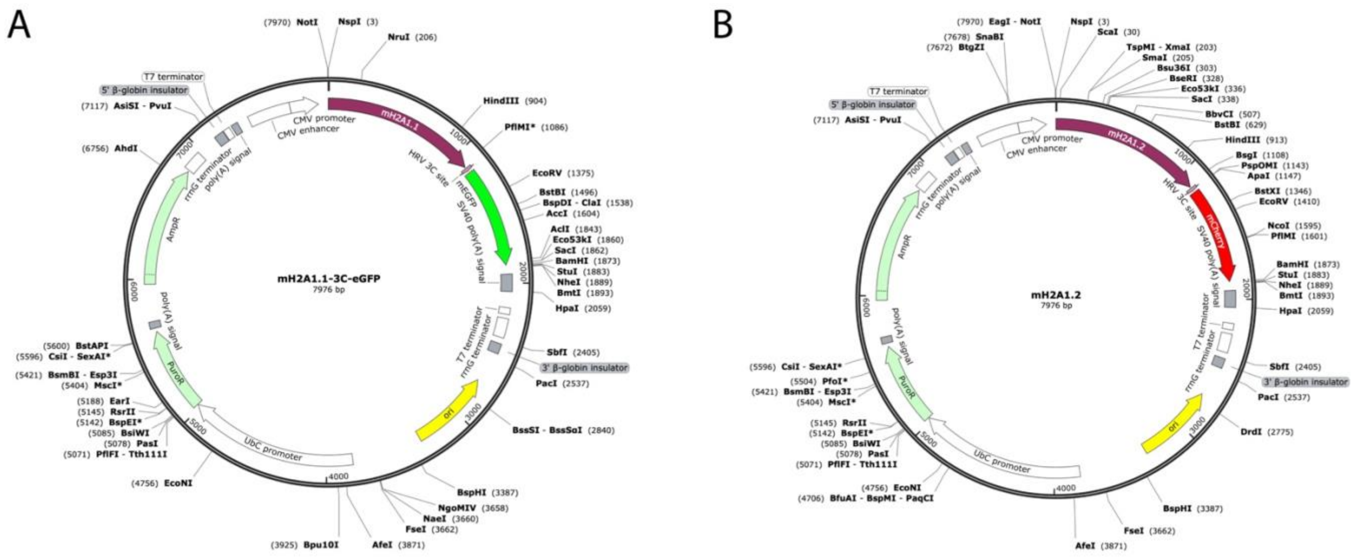

Supplementary Figure 2. Linear correlation between the percentage of mH2A1 positive cells and the histological cellularity in the biopsy of MDS patients. The 3 MDS cases negative for mH2A1 expression were excluded from the graph.

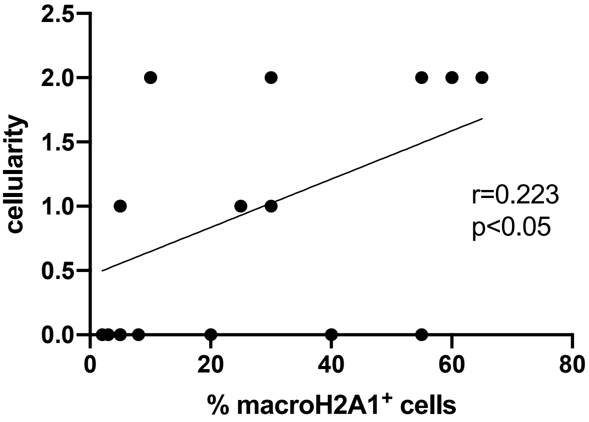

Supplementary Figure 3. A-B. Western blot analysis of mH2A1.1 and mH2A1.2 after 48 and **C.** TLR4 after 72h from starting transfection. H3 or GAPDH proteins were used as total protein loading reference. For analysis the optical density of the bands was measured using Scion Image software. **D.** Analysis of NFkB nuclear translocation by immunofluorescence. Representative immunofluorescence pictures of CTL, mH2A1.1-OE and mH2A1.2-OE cells are reported and quantification of nuclear NFkB MFI was graphed. Scale bar: 10 μm. **E.** Western blot showing specific depletion of mH2A1 protein after 72 from starting silencing. H3 was used as loading control. Si-mH2A1: HS-5 cells transfected using siRNA directed against mH2A1. **F.** Analysis of TLR4 expression after 72h upon transfection. GAPDH was used as loading control. All the data are presented as means ± SD of three independent experiments. *p<0.05; **p<0.01; ***p<0.001; ****p<0.0001.

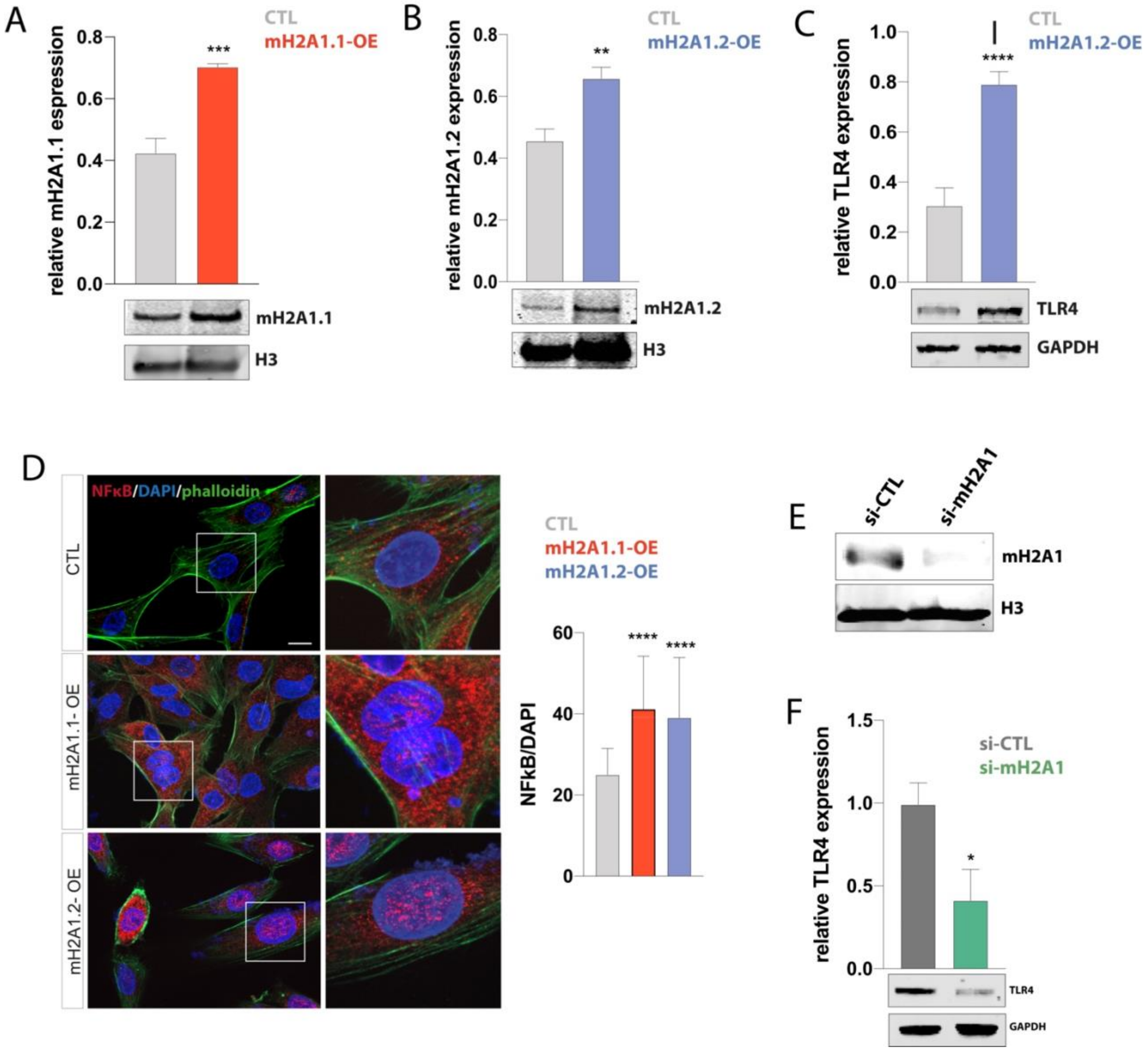

Supplementary Figure 4. A-B. Western blot analysis of PARP1 and AHCY. H3 or GAPDH proteins were used as total protein loading reference. For analysis the optical density of the bands was measured using Scion Image software. **C.** Representative immunofluorescence pictures of CTL and mH2A1.1-OE cells for CBX3/HP1γ. The protein MFI was analyzed and quantification was represented as fold change over CTL. Scale bar: 20 µm. **D.** Abundance of metabolites evaluated by HPLC analysis in HS-5 cells overexpressing mH2A1.1. Cells were transfected for 72h by using mH2A1.1-CT-MYC plasmid or empty vector. Data are shown as $\log_2$ FC (fold change) over CTL of four separate experiment for condition **E.** ROS levels were quantified after 72h from starting transfection evaluating oxidation of DCF by flow cytometry. Data are presented as means ± SD of three independent experiments. *p<0.05; **p<0.01

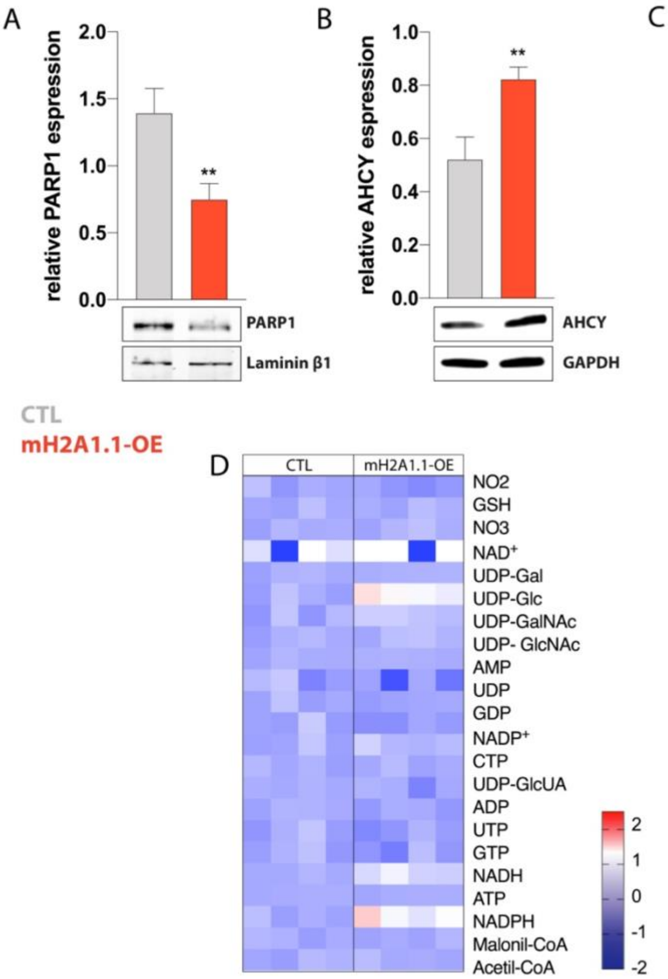

Supplementary Figure 5. A. Flow cytometric analysis of the absolute number of healthy CD34^{+} cells cultured alone or derived from cocultures with CTL-MSC feeder or mH2A1.2-OE MSCs for 5 days. **B.** Frequencies for CFU-BFU, CFU-GM and CFU-GEMM were calculated after 14 days after co-cultures were stopped. Data represent mean ± SD of three independent experiments performed in duplicate.

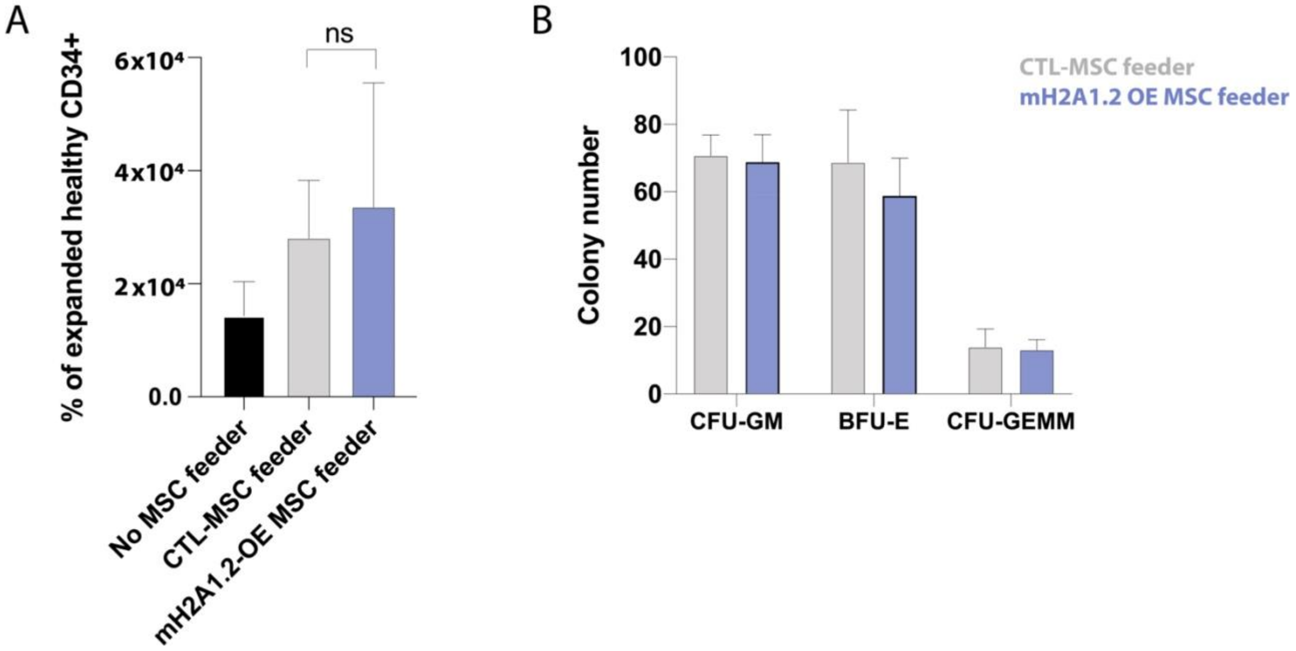
A
ns
6x10⁴
4x10⁴
2x10⁴
0.0
% of expanded healthy CD34+
No MSC feeder
CTL-MSC feeder
mH2A1.2-OE MSC feeder
B
100
80
60
40
20
0
Colony number
CTL-MSC feeder
mH2A1.2 OE MSC feeder
CFU-GM
BFU-E
CFU-GEMM
