# Supplementary material for: MacroH2A1.1 as a crossroad between epigenetics, inflammation and metabolism of mesenchymal stromal cells in myelodysplastic syndromes
Source: Cell Death Dis. 2023 Oct 18;14(10):686. doi: 10.1038/s41419-023-06197-x (PMC10584900; doi:10.1038/s41419-023-06197-x)

mH2A1.1 protein (40 kDa) (SF.2A)
CTL and mH2A1.1-OE

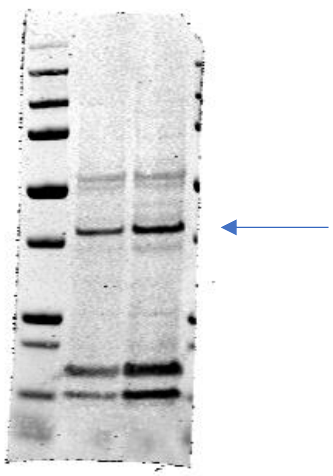

H3 protein (17 kDa) (SF.2A)
CTL and mH2A1.1 OE

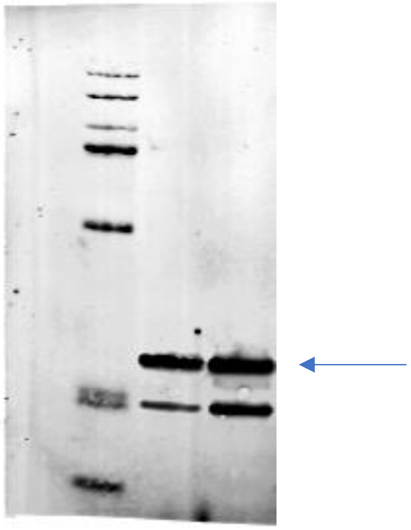

TLR4 (95 kDa) and β-Actin (42 kDa) proteins (Fig.2I)

CTL and mH2A1.1-OE

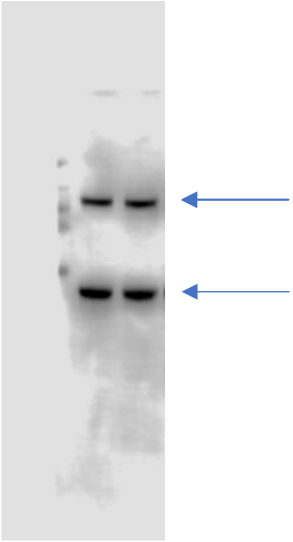

NFkB (65 kDa) protein (Fig.2J)
CTL and mH2A1.1-OE

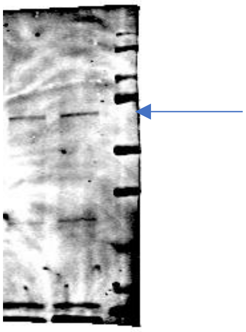

Laminin β1 (200 kDa)
CTL and mH2A1.1-OE

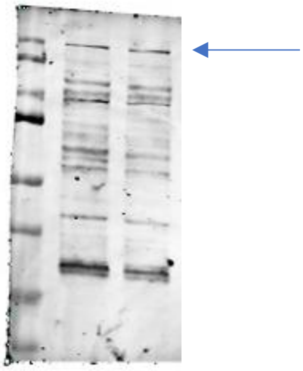

mH2A1.2 (40 kDa) and H3 (17 kDa) proteins (SF.2B)
CTL and mH2A1.2-OE

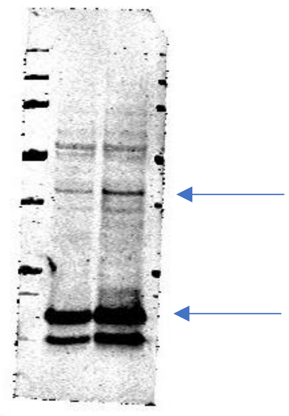

TLR4 (95 kDa) and GAPDH (35 kDa) proteins (SF.2C)
CTL and mH2A1.2-OE

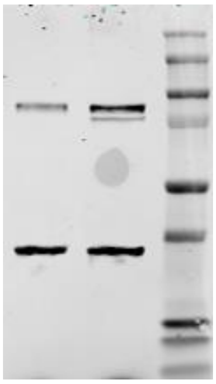

yH2AX protein (15 kDa) Fig.2M
CTL and mH2A1.1-OE

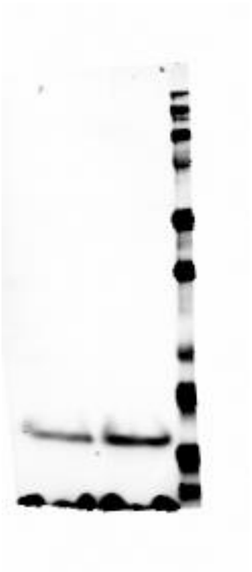

H3 protein (17 kDa) Fig.2M
CTL and mH2A1.1-OE

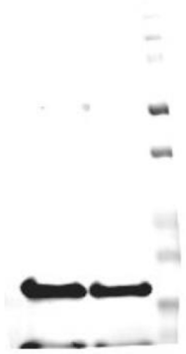

mH2A1(40 kDa) and H3 (17 kDa) proteins (SF3E)
si-CTL and si-mH2A1

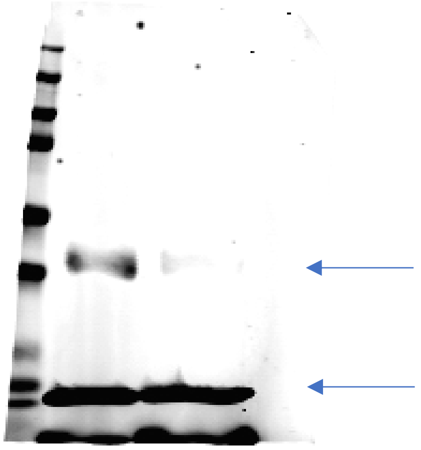

TLR4 (95 kDa) and GAPDH (35 kDa) proteins (SF.3F)
si-CTL and si-mH2A1

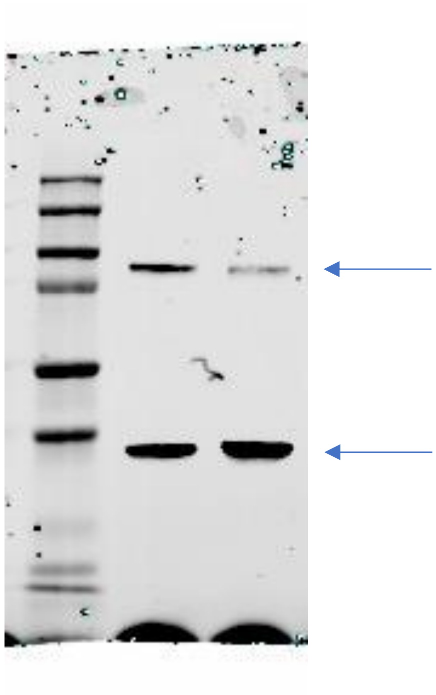

PARP1 protein (116 kDa)  (SF4A)
CTL and mH2A1.1-OE

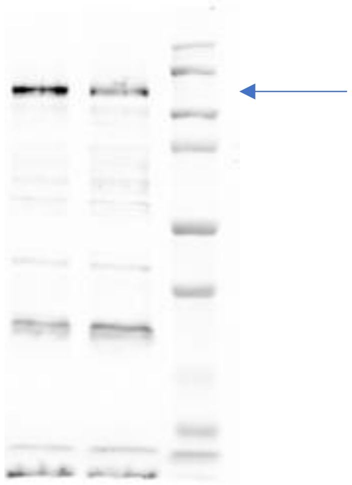

Laminin β1 (200 kDa) (SF4A)
CTL and mH2A1.1-OE

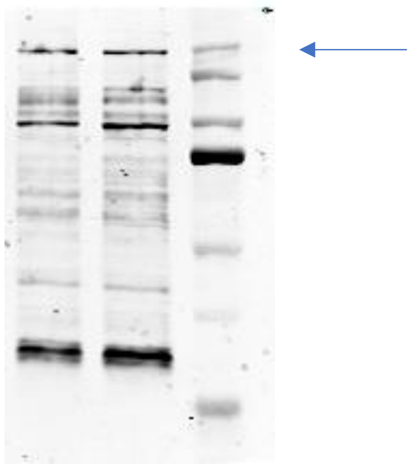

AHCY protein (48 kDa)  (SF4B)
CTL and mH2A1.1-OE



GAPDH protein (35 kDa)  (SF4B)
CTL and mH2A1.1-OE

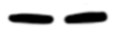

H3K9me3 protein (15 kDa) (Fig.4D)
CTL and mH2A1.1-OE

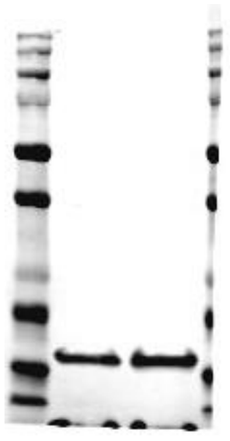

H3 protein (17 kDa) (Fig.4D)
CTL and mH2A1.1-OE

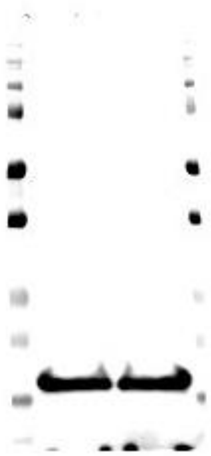

Laminin β1 (200 kDa) and LDHA (36 kDa) (Fig.4J)
CTL and mH2A1.1-OE

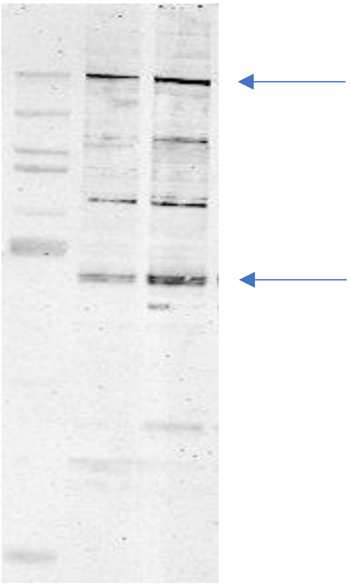

mH2A1.1 protein (40 kDa) (Fig.6A)
CTRL-5uM-10uM AZA

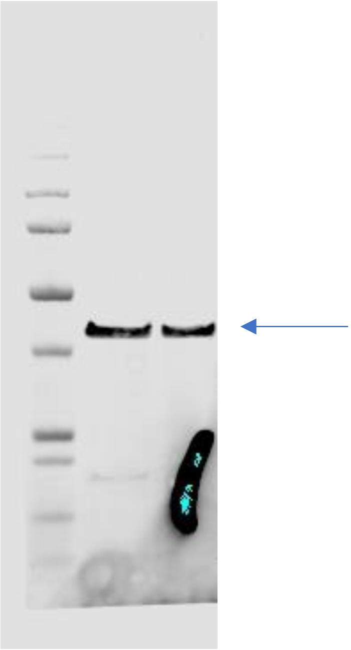

H3 protein (17 kDa) (Fig.6A)
CTRL-5uM-10uM AZA

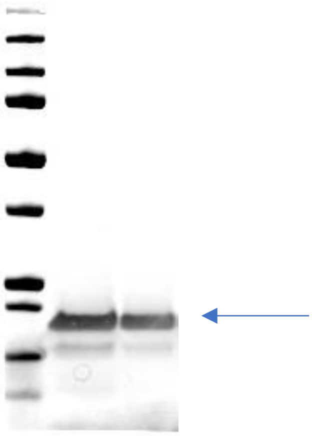

GAPDH protein (36 kDa) (Fig.6B)
CTRL-5uM-10uM AZA

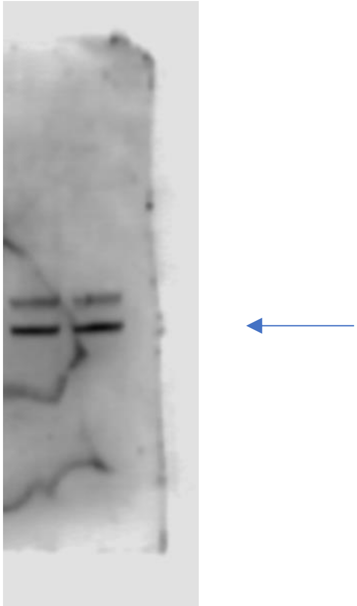

TLR4 (95 kDa) (Fig.6B)
CTRL-5uM-10uM AZA

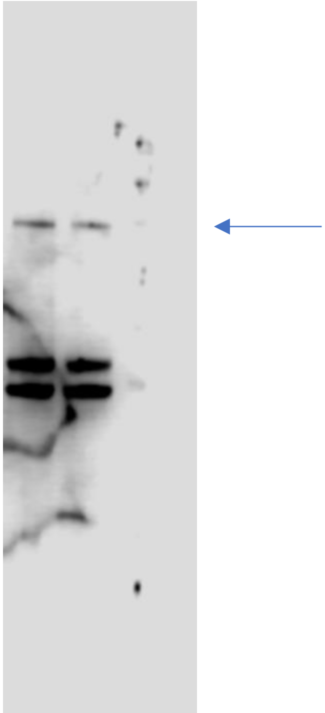

Supplement: Supplementary file 2 — Original Data File [file 41419_2023_6197_MOESM2_ESM.pdf]
